# AI-big data analytics for building automation and management systems: a survey, actual challenges and future perspectives

**DOI:** 10.1007/s10462-022-10286-2

**Published:** 2022-10-15

**Authors:** Yassine Himeur, Mariam Elnour, Fodil Fadli, Nader Meskin, Ioan Petri, Yacine Rezgui, Faycal Bensaali, Abbes Amira

**Affiliations:** 1 Department of Architecture & Urban Planning, Qatar University, Doha, Qatar; 2 College of Engineering and Information Technology, University of Dubai, Dubai, UAE; 3 Department of Electrical Engineering, Qatar University, Doha, Qatar; 4 School of Engineering, BRE Institute of Sustainable Engineering, Cardiff University, Wales, UK; 5 Department of Computer Science, University of Sharjah, Sharjah, UAE; 6 Institute of Artificial Intelligence, De Montfort University, Leicester, UK

**Keywords:** Artificial intelligence, Big data analytics, Building automation and management system, Deep learning, Evaluation metrics, Computing platforms

## Abstract

In theory, building automation and management systems (BAMSs) can provide all the components and functionalities required for analyzing and operating buildings. However, in reality, these systems can only ensure the control of heating ventilation and air conditioning system systems. Therefore, many other tasks are left to the operator, e.g. evaluating buildings’ performance, detecting abnormal energy consumption, identifying the changes needed to improve efficiency, ensuring the security and privacy of end-users, etc. To that end, there has been a movement for developing artificial intelligence (AI) big data analytic tools as they offer various new and tailor-made solutions that are incredibly appropriate for practical buildings’ management. Typically, they can help the operator in (i) analyzing the tons of connected equipment data; and; (ii) making intelligent, efficient, and on-time decisions to improve the buildings’ performance. This paper presents a comprehensive systematic survey on using AI-big data analytics in BAMSs. It covers various AI-based tasks, e.g. load forecasting, water management, indoor environmental quality monitoring, occupancy detection, etc. The first part of this paper adopts a well-designed taxonomy to overview existing frameworks. A comprehensive review is conducted about different aspects, including the learning process, building environment, computing platforms, and application scenario. Moving on, a critical discussion is performed to identify current challenges. The second part aims at providing the reader with insights into the real-world application of AI-big data analytics. Thus, three case studies that demonstrate the use of AI-big data analytics in BAMSs are presented, focusing on energy anomaly detection in residential and office buildings and energy and performance optimization in sports facilities. Lastly, future directions and valuable recommendations are identified to improve the performance and reliability of BAMSs in intelligent buildings.

## Introduction

### Preliminary

Building automation and management systems (BAMSs) are intelligent systems of both hardware and software, connecting heating ventilation and air conditioning system (HVAC) systems, lighting, security, and other systems to communicate on a single platform. That said, BAMSs deliver crucial information to operators and/or users on the operational performance of buildings, which aim at promoting energy efficiency and optimizing water consumption, enhancing the safety and comfort of the occupants, reducing maintenance costs, extending the life cycle of the utilities, etc (Ippolito et al. 2014). This is possible by networking a plethora of sensors and components responsible for the monitoring and operation of mechanical, security, fire, lighting, HVAC and humidity control and ventilation systems (Su and Wang 2020).

With the broad utilization of information and communication technologies (ICTs), sensing and measurement technologies along with the cloud computing, big data storage and data analytics, conventional BAMSs are being revolutionized. Vast quantities of building automation and management data are produced, gathered and saved (Sardianos et al. 2020; Himeur et al.). This has offered an excellent opportunity for implementing big data mining and analysis in BAMSs. In this context, as the quantity of data collected in BAMSs is enormous, the ”big data” phenomena is surfacing this field and revolutionizing the way we manage data by using AI-big data analytics tools (Quinn et al. 2020, Himeur et al.). Accordingly, with advanced sensing and metering technologies in BAMSs, data split into multiple modalities and many variables can create a comprehensive source of information to analyze. This allows for more targeted analysis, but also means that more powerful, intelligent, and sophisticated tools are needed to identify the most enormous patterns/variables (Muntean et al. 2021). As a consequence, the big data analytics market in the building energy sector is expected to grow at a Compound annual growth rate (CAGR) of 11.28%, during the forecast period, 2021–2026.^1^ Data collection in the building industry is becoming all-embracing. This wealth of big data allows informed data-driven decision-making by designers, facilities managers, and owners during building design, operation, and retrofit (Berger et al.). On the other hand, for existing or outdated buildings to make full use of the services offered by the flourishing data analytics market, the necessary enhancement to the existing system for deploying the new technology must be addressed and sorted out (Varlamis et al. 2022). The main challenges of gathering and analyzing data of old buildings are the outdated technologies and the conventional error-prone data collection means (Jia et al. 2019; Al Dakheel et al. 2020). Nevertheless, data analytics can assist in designing and implementing the new system adaptation and existing system renovation (Elnour et al. 2022).

Besides, it is of utmost importance to know the current state of AI-based building automation before presenting the actual study concerning user input, demand, response, energy-saving, and automation. In this respect, it is obvious that AI adds new dimensions to building automation environments by enabling autonomous data analysis for operation optimization. Therefore, many AI-based contributions have recently emerged as key solutions for (i) predicting building occupancy, (ii) forecasting thermal comfort, (iii) boosting energy saving, and (iv) enabling demand-side response (Himeur et al. 2020). Additionally, as mentioned in previous studies, (O’Grady et al. 2021) people can spend up to 90 percent of their lives in buildings; this highlights the importance of user input, behavioral data, and behavioral analytics for optimizing and automating building operations. To that end, a significant research effort is ongoing to develop AI-based behavioral change technologies to promote energy saving in residential and office buildings (Sayed et al., Varlamis et al. 2022), understand consumers demand patterns for successful demand response development (Cruz et al. 2021; Pratt and Erickson 2020), optimizing occupants’ thermal comfort (Zheng et al. 2022), transforming water management (Doorn 2021), improving fault detection and diagnosis (Yun et al. 2021), etc. Moreover, AI-based big data analytics are contributing to building automation by making BAMSs self-learning, self-configuring and self-diagnosing, and self-commissioning (Katipamula 2019). Additionally, using AI-based analytics can adapt existing building systems to promote the deployment of BAMSs with fewer investments from building owners.

From another hand, as AI models are very competent to learn common human error patterns, their use in big data analytics is significant. They can (i) detect and resolve possible flaws in datasets, (ii) learn by watching how the operators and users interact with the analytics programs, and identify anomalies and surface unexpected insights from large-scale datasets fast (Mahmud et al. 2020; Diamantoulakis et al. 2015). In this context, AI models assist operators and users of BAMSs to perform the different tasks related to the big data cycle, among them the operations of collecting, pre-processing, aggregating, storing, analyzing and extracting various kinds of features (Hu and Vasilakos 2016; Bode et al. 2019). Moving on, the integration of AI-big data analytics can (i) optimize energy and operational efficiency, (ii) automate monitoring and control through wireless platforms, (iii) provide quick and better decision making, (iv) smartly control the facility and reduce risk failures, (v) lower life cycle costs, and (vi) increase safety and security measures with ease (Aghemo et al. 2014; Zhou and Yang 2016; Aste et al. 2017).

### Paper contributions

Due to the importance of using AI-big data analytics in BAMSs, a plethora of works have been proposed to (i) address different challenges, (ii) improve and automate building operation, and (iii) optimize building user experience. In addition, different reviews have been introduced to discuss the advances made in this research topic, such as (Zhang et al. 2021; Molina-Solana et al. 2017; Zhao et al. 2020). However, most of them have only focused on addressing one task at a time, e.g., energy management, rather than covering multiple BAMS tasks together (e.g., water management, occupancy detection, comfort optimization, fault diagnosis and anomaly detection (FDAD), etc.) (Sun et al. 2020; Wang et al. 2021; Fan et al. 2018). To that end, we present in this paper a comprehensive systematic survey reflecting the latest developments in the field of AI-big data analytics and their utilization in BAMSs from different perspectives. For example, Zhang et al. (2021) discuss sensor impact verification and evaluation for FDAD in energy systems, while Molina et al. (2017) review the contributions of data science for building energy management issues. Moving on, data mining strategies used for building energy management are overviewed in Zhao et al. (2020). Similarly, in Sun et al. (2020), data-driven techniques for energy prediction in buildings are described. Besides, Wang et al. (2021) focus on studying the practical problems related to implementing ML models for building energy efficiency. It also investigates the commitment of existing studies to comfort and energy saving (i.e., Save energy with/without compromising thermal comfort). Moreover, in Fan et al. (2018), unsupervised data mining methodologies for energy efficiency improvement are analyzed. Lastly, in Pinto et al. (2022), Pinto et al. discuss the roles of transfer learning integration for smart buildings and systems.

To that end, we present in this paper a comprehensive survey reflecting the latest developments in the field of AI-big data analytics and their utilization in BAMSs from different perspectives. Thus, we first introduce a generic taxonomy for classifying AI-big data analytics frameworks based on various criteria, including the learning method, building environment, computing platform, and application or challenge addressed. Typically, an overview of existing works and discussions is presented, highlighting some of the challenges, limitations, and shortcomings. Then, three case studies are presented illustrating the use of AI-big data analytics for critical concerns in the buildings sector, that is, energy efficiency and management, to provide the reader with insight into real-world applications. The optimization of energy consumption in buildings has been a hot research topic recently^2^ in terms of efficient planning, proper management, and preventive maintenance. Lastly, future directions to ease the use of AI-big data analytics models in BAMSs and improve their feedback are derived. To summarize, the contributions of the presented work are manifold:

- Providing a thorough review covering the general use of AI-big big data analytics in BAMSs and shedding light on their increasing importance for developing efficient and smart BAMSs.
- Presenting a well-designed taxonomy of existing AI-big data analytics frameworks, which helps in understanding intriguing relationships between various concepts and variables in the field. Different criteria have been adopted when analyzing existing frameworks, including the learning method, building environment, computing platform, application, etc.
- Conducting a critical analysis and discussion to (i) extract diverse relevant lessons that are learned from overviewed works; and (ii) highlight open issues and current challenges, among them data scarcity, data benchmarking, security and privacy, scalability and interoperability and real-time big data intelligence.
- Presenting three case studies that describe the use of AI-big data analytics in BAMSs for buildings energy management and optimization, such that the first two case studies demonstrate unsupervised and supervised energy anomaly detection strategies in residential and office buildings, and the third one is about energy and performance optimization in sports facilities.
- Deriving a set of future research and development directions that attract considerable interest in the near and far future, and help in improving the performance and reliability of BAMSs.

Table 1 outlines some of the main differences between the actual review and other survey studies. It also sheds light on some of the main contributions addressed by this review compared to the others in terms of overviewed resources (i.e., ML tools and computing platforms), application scenarios, discussed challenges (i.e., security issues), evaluation metrics, case studies, and proposed future directions (i.e., multimodal data analysis, in-situ sensor calibration in BAMSs, smart building digital twins, blockchain edge analytics, etc.).

**Table 1:** Comparison of the proposed survey’s contributions against other existing related related review studies

	ML tools	Computing platforms	Application scenarios	Security issues	Evaluation metrics	Case studies	Future directions			
							Multimodal data analysis	Blockchain	Edgeanalytics	3D point clouds
Zhang et al. (2021)	✗	✗	FDAD	✗	✗	✗	✗	✗	✗	✗
Molina-Solana et al. (2017)	✓	✓	Energy management	✓	✗	✗	✗	✗	✗	✗
Zhao et al. (2020)	✓	✓	Energy management	✗	✗	✗	✗	✗	✗	✗
Sun et al. (2020)	✓	✗	Energy prediction	✗	✗	✗	✗	✗	✗	✗
Wang et al. (2021)	✓	✗	Energy efficiency	✗	✗	✗	✓	✗	✗	✗
Fan et al. (2018)	✓ (unsupervised)	✗	Energy efficiency	✗	✗	✗	✗	✗	✗	✗
Pinto et al. (2022)	✓ (transfer learning)	✗	Load prediction, system control, occupancy detection, building dynamics	✗	✓	✗	✗	✗	✗	✗
Our paper	✓	✓	Energy management FDAD, IEQ, security and safety, occupancy detection and water management	✓	✓	✓	✓	✓	✓	✓

### Review methodology

A well-established review methodology is adopted in this paper, where we first conduct a comprehensive literature search in the most popular scientific databases, including Scopus, Elsevier, Wiley, and IEEE. Following, most of the works that deal with the use of AI-big data analytics for BAMSs are included in this study. Many keywords and their combination are then used in the search, e.g., ”building automation and management systems”, ”big data analytics”, ”artificial intelligence”, ”machine learning”, ”deep learning”, ”transfer learning”, ”energy prediction in buildings using machine learning”, ”thermal comfort in building using machine learning”, ”fault diagnosis and anomaly detection in buildings”, ”security in building automation and management systems”, etc. Therefore, research studies introduced between January 2015 and February 2022 are discussed in this framework. This period has arbitrarily been selected to evaluate the recent and pertinent contributions. Typically, this framework discusses English-written peer-reviewed journal articles, conference proceedings papers, and book chapters. The selection process adopted in this review relies on adhering to the specifications of the PRISMA (Moher et al. 2009), which is a practical and efficient approach for writing survey studies. Concretely, a search was performed for the last seven years (January 2015–February 2022). To eliminate duplicate references, a reference manager software was utilized, and only the remaining frameworks have then been considered after filtering them by their titles, keywords, and abstracts.

In addition to reviewing existing AI-big data analytics contributions for BAMSs, three case studies are also included in this article to provide the reader with more explanations about using AI tools in tackling the buildings’ energy consumption question in terms of (i) unsupervised energy anomaly detection, (ii) supervised energy anomaly detection, and (iii) energy and performance optimization for sports facilities.

### Organization of the paper

The rest of this paper is structured as follows. Section 2 highlights the significant advances made in the development of BAMSs. Section 3 provides an overview of the interdisciplinary AI-big data analytics research in BAMSs following a well-defined taxonomy. Section 4 evaluates and critically analyses overviewed frameworks to identify the open issues and current challenges. Section 5 presents three case studies that describe the use of AI-big data analytics in BAMSs for energy anomaly detection in residential and office buildings, and energy optimization in sports facilities. Moving on, Sect. 6 presents the future directions for improving the performance of BAMSs. Finally, conclusions and significant findings are summarized in Sect. 7.

## Evolution of BAMSs

In the last decades, BAMSs have been rapidly developed; indeed, from the 1950s to 1990s, they have been transformed from pneumatics to electronics then open protocols (e.g. BACnets). Moving forward, with the digitization era, BAMSs have further progressed by (i) integrating more powerful and smart technologies, (ii) becoming easier to implement in different kinds of buildings, and (iii) using high-quality softwares to aid the users in getting the most pertinent information from their buildings. Indeed, digitization has significantly accelerated with the launch of smartphones, where these devices have abruptly replaced mobile communicators, cell phones, etc. and become more practical in many real-world applications as they deliver different benefits, such as supporting apps.

### Progress made during the two last decades

**Fig. 1:**
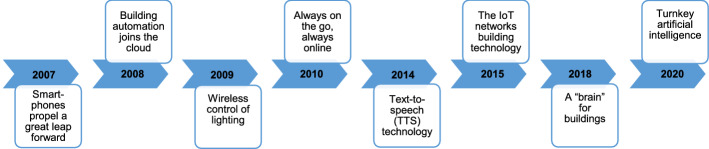
The significant progress made in the development of BAMSs during the last two decades

We briefly described in this section the most significant achievements made in the development of BAMSs during the last two decades, which can be summarized as portrayed in Fig. 1. Typically, in 2008, it became possible to virtualize BAMSs in data centers, and hence receives greater security and availability and enables more flexible access to buildings’ data. In 2009, WiFi was integrated to BAMSs to help in flexibly and remotely monitoring appliances in households and commercial centers (Wang 2009). Following, in 2010, due to the growing utilization of smartphones and tablet computers in smart city applications to control smart and location-based products, BAMSs have been also positively influenced through developing more sophisticated and portable BAMSs solutions (Aste et al. 2017).

By 2014, audio files had been deployed in BAMSs using the text-to-speech (TTS) technology. The latter enabled to support preventive inspections and maintenance, work contracts, service requests, work contracts, equipment audits, etc (Mundt et al. 2014). In 2016, IoT began to significantly influence the society after that IoT devices found their way into the building management sector, where six billion devices were installed and more than 31 billion were expected in 2020 (Aste et al. 2017). Explicitly, the importance of automation has been increased in both existing and new buildings. Following, in 2018, commercial buildings have progressively evolved into smart buildings and routines have been improved and/or automated in BAMSs, resulting in enhanced comfort and efficiency (Markoska and Lazarova-Molnar 2018; Lee and Karava 2020). Lastly, in 2020, AI joined the spectrum of BAMSs to help in early fire detection, and energy demand prediction. Moreover, it becomes possible to identify behavioral change through the analysis of real-time data. Thus, BAMSs learn from experiences and historical data for automatically adjusting the indoor conditions (Yaïci et al. 2021).

### Big data sources

**Fig. 2:**
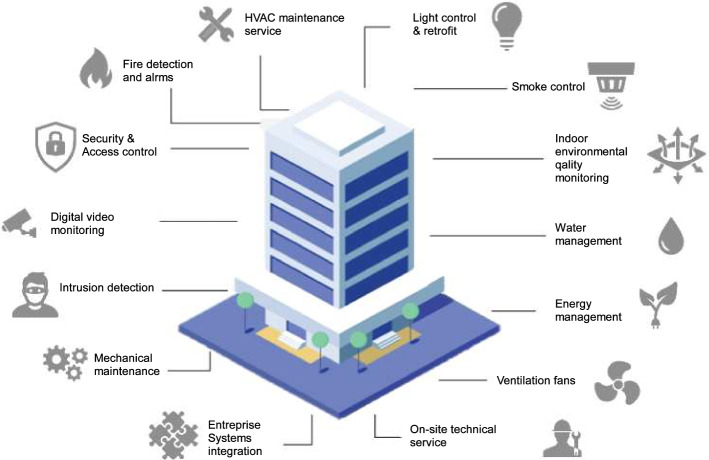
Principal services of a BAMS system

This section discusses and describes the essential sources of heterogeneous big data used to implement AI-big data analytics in BAMSs. Indeed, developing and accelerating the advance and deployment of BAMSs require the installation of a large number of smart sensors, smart meters, and other measurement devices in the different parts of each building, which helps in (i) increasing the observability of its transient and dynamic events, and (ii) gather actual data related to the diverse functionalities of the building. This will later help the AI-big data analytics in accurately analyzing this data and extracting pertinent features and therefore facilitating the operation and monitoring of all building technology, especially in larger buildings. Figure 2 portrays the overall architecture of a BAMS and its principal data sources. The control module is the central brain of the BAMS, and most of the controllers are built using the industry standard BACnet protocols in addition to Konnex (KNX); an open communication standard for commercial and domestic building automation, LonWorks; a standardized bus system used in centralized and decentralized building automation control (Merz et al. 2018), and Modbus; a network communication protocol for connecting electronic equipment in industrial automation systems. Overall, a BAMS can provide various services to control (i) heating and cooling, (ii) lighting, (iii) security, (iv) access control, (v) fire and life safety, and (vi) elevators and escalators.

## Overview of AI-big data analytic frameworks

### Overall taxonomy

**Fig. 3:**
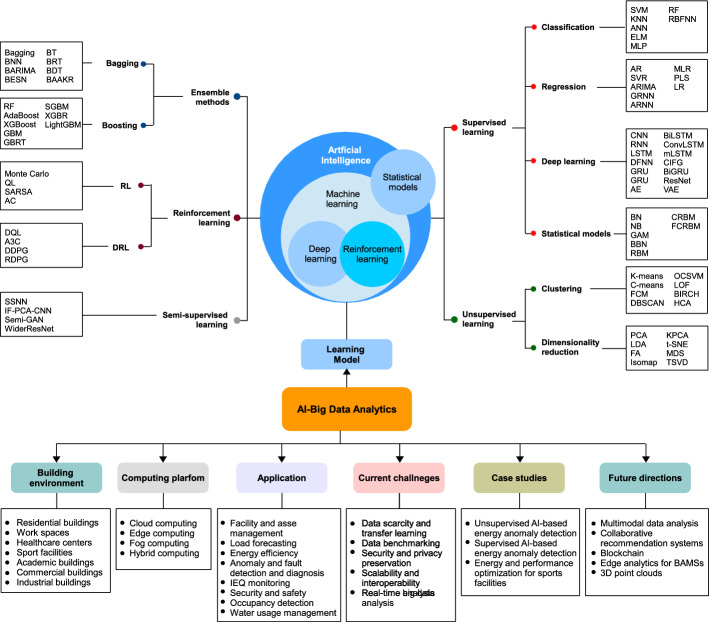
Taxonomy of existing AI-big data analytics frameworks

To understand the challenges related to AI-big data analytics in BAMSs, it is essential to perform a generic taxonomy of existing AI-big data analytics techniques used for monitoring the smart buildings. Specifically, Fig. 3 provides a structured analysis framework that helps in overviewing existing techniques and shedding the light on the organization of the presented framework.

### AI-learning process

The first step in any AI process is system learning. This can take four primary forms: supervised learning, unsupervised learning, semi-supervised, and reinforcement learning. In this section, we present an overview of existing AI learning architectures used to improve the performance of BAMSs.

#### Unsupervised learning (U)

Unsupervised learning learns from raw data without prior knowledge and mainly deals with unlabeled datasets. Although it does not need to annotate data as supervised learning, the learning phase can be more computational as all the possibilities are checked. The accuracy is lower since there are no corresponding outputs (labels) (Himeur et al. 2021).

##### U1. Clustering

It is a category of ML algorithms used for separating data (e.g. energy consumption observations, ambient conditions, etc.) into different classes or clusters following a specific goal. Clustering algorithms usually pertain to one of the following groups, i.e. hybrid, fuzzy-based, model-based, and density-based approaches. Using the clustering process facilitates the classification tasks when dealing with various problems, such as anomaly detection of energy consumption, indoor environmental quality (IEQ) monitoring and detection of pollutants, detection of abnormal water consumption, etc.

K-means, C-means and fuzzy C-means (FCM) were among the most investigated clustering approaches. They have been applied for non-intrusive load monitoring (NILM) and appliance identification (Ji et al. 2019; Zhang et al. 2020), energy performance evaluation and ranking in working spaces (Sun and Yu 2021), energy efficiency assessment in industrial buildings (Liu et al. 2018), building management and identification of operating anomalies (when analyzing electricity, gas and water consumption) (Akil et al. 2019), IEQ monitoring (Dogruparmak et al. 2014; Cao et al. 2020; Alghamdi et al. 2020; Roger Rozario 2021), energy forecasting (Tian et al. 2020; Chen et al. 2020; El Motaki et al. 2021), and data sampling for better visualization (Qin and Zhang 2017).

In Culaba et al. (2020), an energy prediction model is introduced using a k-means model to cluster data, and a support vector machine (SVM) is employed to forecast energy consumption. In Himeur et al. (2021), three clustering algorithms, namely one-class support vector machine (OCSVM), density-based spatial clustering of applications with noise (DBSCAN), and local outlier factor (LOF), are used to detect anomalous energy consumption in households by analyzing energy footprints. Besides, in Afaifia et al. (2021), hierarchical cluster analysis (HCA) is implemented to model residential energy consumption and promote energy efficiency. Clustering-based techniques have been used in BAMSs because of their simplicity and relatively computational efficiency. Also, clustering models generally have few parameters to tune. However, they have different limitations that affect their applications in BAMSs, among them the manual selection of the optimal *K*, dependency on initial values, troubles to cluster data with varying densities and sizes, the need for scaling as the number of dimensions increases, etc. (Li et al. 2018).

##### U2. Dimensionality reduction

In diverse ML tasks, dimensionality reduction techniques can be employed to classify data while promoting low computational costs as they first remove irrelevant observations. Accordingly, a plethora of frameworks have been proposed in the literature to explore the applicability of dimensionality reduction schemes in BAMSs. That includes the principal component analysis (PCA), factor analysis, linear discriminant analysis (LDA), quadratic discriminant analysis (QDA), and multiple discriminant analysis (MDA), isometric feature mapping (Isomap) (Liu et al. 2020), kernel principal component analysis (KPCA) (Abba et al. 2020), t-distributed stochastic neighbor embedding (t-SNE) (Zhan et al. 2020; Lopes et al. 2020), multidimensional scaling (MDS) (Wang 2020, and truncated singular value decomposition (TSVD) (Kalantzis et al. 2021).

For instance, PCA has been utilized for early fault detection and classification (Li and Wen 2014; Cotrufo and Zmeureanu 2016; Chen and Wen 2017; Swiercz and Mroczkowska 2019), IEQ (Mansor et al. 2021), energy consumption prediction (Sha et al. 2019), occupancy detection in buildings (Pal et al. 2019), etc. Moving on, LDA has been employed for thermal comfort evaluation (Gładyszewska-Fiedoruk and Sulewska 2020), sensor-based occupancy detection (Fayed et al. 2019). Using dimensionality reduction in BAMS has gained attention because it can: (i) reduce the storage space and time needed to classify recorded data, (ii) improve the interpretation of the ML models’ parameters by removing multicollinearity, and (iii) simplify data visualization (Al-Kababji et al. 2022). However, dimensionality reduction models have some disadvantages. For example, (i) this can result in relevant data loss, (ii) finding linear correlations between variables (as PCA does) can be no appropriate in some scenarios, and (iii) some dimensionality reduction models can fail in classifying variables if the covariance and mean are not sufficient to represent datasets (Himeur et al. 2021; Abdulhammed et al. 2019).

#### Supervised learning (S)

Supervised learning is applied for the case of labeled energy datasets. Despite its high performance, the necessity of labeled data causes some difficulties in real-world applications.

##### S1. Classification

It refers to conventional ML models that attempt to derive some conclusions from the input data given in the training process, and hence aim at predicting the class labels/categories for a new set of data. Classification models have widely been deployed in existing BAMS based big data analytics frameworks to perform different tasks, e.g. energy forecasting, energy balancing, IAQ monitoring, energy optimization, fault and anomaly detection. Typically, SVM, K-nearest neighbors (KNN) (Valgaev et al. 2017), decision tree (DT) (Yu et al. 2010), artificial neural network (ANN) (Moon et al. 2019), multi-layer perceptron (MLP) (Haidar et al. 2019), extreme learning machine (ELM) (Salerno and Rabbeni 2018) and logistic regression (LR) (Rehman et al. 2020) are among the famous classification models deployed in BAMSs. Classification models have been used in BAMSs since they are simple to understand, fast and efficient. In addition, they can excel in classifying different kinds of BAMS data if accurately labeled datasets are used in the training process. However, they have a set of limitations. For instance, SVM models are not adequate for non-linear problems, and their performance does not improve if the number of features increases while the number of neighbors ”K” is manually selected in KNN. Moreover, poor results are usually obtained with DT algorithms on small datasets, and overfitting can quickly occur (Himeur et al. 2021).

##### S2. Regression

It is based on the identification of the relation between two or more energy consumption observations for producing a set of model parameters, that help in predicting and classifying them for different purposes, including energy prediction, anomaly detection, security and privacy preservation, etc. Diverse regression models have been proposed to analyze BAMSs’ data, e.g. support vector regression (SVR) (Zhong et al. 2019), linear regression (LR), auto-regressive (AR) models, regression tree (RT) and regression fitting (RFT). Regression models have gained popularity in smart buildings and smart energy systems because most of them are easy to implement and interpret, and efficient to train. Also, they perform remarkably well for linearly separable data. However, it is worthy to note that regression models involve complicated and lengthy procedures of analysis and calculations in addition to assuming the existence of linearity between the dependent and independent variables, which is not always the case in real-world applications (Bilous et al. 2018).

##### S3. Deep neural networks (DNN)

It is a subclass of ML that is principally a NN including more than two layers. These DNNs aim at simulating the behavior of the human brain ”albeit far from matching its ability”, which allows them to learn from large-scale datasets. In addition to the capability of NNs with a single layer for making approximate predictions, DNNs have further benefits via (i) optimizing and refining the classification accuracy when additional hidden layers are considered, and (ii) identifying the most informative features of the data.

DNNs have become the state-of-the-art methods in various ML-based domains, similarly in BAMSs, they are attracting greater attention. They have widely used for energy forecasting, this is the case of recurrent neural networks (RNN), long short-term memory (LSTM) (Gao et al. 2019; Wang et al. 2020), gated recurrent unit (GRU) (Lin et al. 2021), bidirectional LSTM (BiLSTM) (Haq et al. 2021; Ishaq and Kwon 2021), convolutional LSTM (ConvLSTM) (Syed et al. 2021), multiplicative LSTM (mLSTM) (Krause et al.), bidirectional GRU (BiGRU) (Khan et al. 2020), coupled input and forget gate (CIFG) (Runge and Zmeureanu), deep feed forward neural networks (DFNN) (Marino et al. 2016), and convolutional neural network (CNN) (Li et al. 2017). Moreover, numerous hybrid models have been built by combining the aforementioned models with other deep learning (DL) architectures, such as CNN-LSTM (Alhussein et al. 2020), CNN-BiLSTM (Wu et al. 2021), partial least square (PLS) CNN-BiLSTM (PLS-CNN-BiLSTM) (Wu et al. 2021), CNN-GRU (Sajjad et al. 2020; Wu et al. 2020), conditional random fields (CRF) and RNN (CRF-RNN) (Wytock and Kolter 2013), DFNN-LSTM (Bashari and Rahimi-Kian 2020), radial basis function neural network-CNN (RBFNN-CNN) (Sideratos et al. 2020), etc.

DL models have also been used for other tasks, including smart IEQ monitoring, where different architectures were investigated, such as LSTM (Liu et al. 2020; Janarthanan et al. 2021), GRU (Ahn et al. 2017; Das et al. 2020), BiLSTM (Ma et al. 2019), CNN (Molinara et al. 2020), residual neural network (ResNet) (Zhang et al. 2020), variational autoencoders (VAE) coupled with CNN (VAE-CNN) (Loy-Benitez et al. 2020a), memory-gated RNN-based autoencoders (MG-RNN-AE) (Loy-Benitez et al. 2020b), sparse autoencoders (SAE) (Loy-Benitez et al. 2020)

Occupancy detection in buildings has also received the attention of the DL community through the use of CNN (Zou et al. 2017), RNN (Zhao et al. 2018), LSTM (Mutis et al. 2020), BiLSTM (Feng et al. 2020). Moreover, as some studies have investigated the use of camera imagery (e.g. thermal cameras) to estimate the number of occupants inside buildings, it was rational to use various CNN backbones, which are widely utilized in image classification or image recognition, among them ResNet (Acquaah et al. 2020), VGGNet (Zou et al. 2017), AlexNet (Acquaah et al. 2020) and GoogLeNet (Tien et al. 2020). Using DL models in BAMSs has become a research hot-spot nowadays because of their robustness to natural variations in the data, which is automatically learned. Additionally, their performance significantly improves with increasing the quantity of training data. However, DL models still face different challenges. Typically, DL algorithms require large-scale training datasets to perform better than other ML models. Moreover, their training is computationally expensive as they are built on complex models. Additionally, DL models require expensive GPUs and cloud data centers to run, which increases their deployment cost (Guo et al. 2018; Himeur et al. 2020).

##### S4. Statistical models

They refer to mathematical models embodying an ensemble of statistical rules used to generate data samples, predict the relationships between one or diverse random/non-random variables or classify them. widely used statistical models include Bayesian networks (BN) (Singh and Yassine 2018), naive Bayes (NB) (Li et al. 2020), generalized additive models (GAM) (Khamma et al. 2020), bayesian belief networks (BBN) (Bassamzadeh and Ghanem 2017), restricted Boltzmann machines (Elsaeidy et al. 2019), conditional restricted Boltzmann machines (CRBM) (Kang et al. 2020) and factored conditional restricted Boltzmann machines (FCRBM) (Hafeez et al. 2020). In BAMSs, they have been used for different tasks, such as selecting the most energy-efficient primary HVAC systems (Tian et al. 2019), building energy and water retrofitting (Bertone et al. 2018), energy forecasting (Huang et al. 2018), assessing energy efficiency (Grillone et al. 2019), NILM (Verma et al. 2019), gas usage prediction (Pathak et al. 2018), IEQ monitoring (Giovanis 2019), etc. While most statistical models are useful for BAMSs as they have deterministic and stochastic components to mathematically describe the functional relationship between inputs and outputs, they also have pitfalls. In this respect, if recorded data is biased or faulty, statistical modeling will be misleading. In addition, these kinds of models are hard to apply to heterogeneous data (Agha and Palmskog 2018).

#### Semi-supervised learning (SSL)

SSL refers to the process of training ML models using a small portion of labeled data along with a large number of unlabeled observations. Then, the ML models should be able to learn and make predictions on new data. It falls between unsupervised learning and supervised learning, which is also considered as a special instance of weak supervision (Van Engelen and Hoos 2020). Although supervised learning techniques are largely utilized in for providing different BMAS services, they can only reach high performance only when they are trained with sufficient labeled data. Otherwise, their performance could drastically decrease if annotated data is insufficient or not accurately labeled. Moreover, annotating data is a challenging, costly, and time-consuming task. In this regard, SSL has been proposed as an alternative solution to address some of these issues.

In BAMS, SSL has been widely used for fault and anomaly detection. For instance, in Fan et al. (2021, 2021), the authors introduce an SSL-based fault detection and diagnosis in air handling units (AHUs) based on a semi-supervised neural network (SSNN), which adopts a self-training strategy. Moving on, in Elnour et al. (2021), Elnour et al. propose an SSL-based data-driven attack detection scheme in HVAC systems to promote security in intelligent buildings. This approach has been developed using an isolation forest and two ML models, i.e. PCA and 1D-CNN (IF-PCA-CNN). While in Li et al. (2021), an SSL-based approach to detect and diagnose chiller faults is presented using a semi generative adversarial network (semi-GAN) model. In the same way, an SSL-based fault identification scheme for building HVAC systems is proposed in Li et al. (2021) using a modified GAN. In Nguyen et al. (2021), an SSL-based load monitoring solution is introduced, which has the ability to (i) augment the data, (ii) transform existing labeled sets, and (iii) train a WiderResNet (the backbone model) on the augmented data. Although SSL is an excellent option for developing AI-big data analytics when labeled data is expensive to obtain, it has some limitations, e.g., the results are not stable, and the performance is lower than that of supervised learning. Typically, the decision boundary might be overstrained if the training dataset does not have the annotated samples required in each class (Lu 2009).

#### Reinforcement learning (RL)

Reinforcement learning is a field in artificial intelligence that involves an agent that develops the knowledge of the best strategy to follow to accomplish a defined objective by trial and error given the interaction with its environment. Besides the RL agent and the environment, the main elements of an RL system are: (i) the policy, which is a function that defines the action taken by the RL agent in a given time step (i.e., state), (ii) the reward, which defines the result of the action taken by the agent due to its interaction with the environment, and intuitively describes the desired behavior of the agent, and (iii) the value, which indicates the long-term desirability of a set of states/actions given the agent’s experience and the likely future rewards (Collins and Cockburn 2020). The agent explores the possible actions to be taken as the learning progresses. Based on the consequence of the actions taken, it opts for actions that maximize the cumulative reward. Reinforcement learning algorithms can be categorized as: (i) traditional RL (TRL) methods in which tabular (i.e. lookup tables) or conventional value function approximation approaches (e.g., coarse coding, ML algorithms) are used; and (ii) deep RL (DRL), which represents the evolution of the traditional methods where DL models (e.g., deep NNs, CNNs, RNNs) are used to approximate the state and/or action value (Wang and Hong 2020).

##### R1. TRL models

It is only efficient to use TRL for simple RL problems where the action-state space can be represented in a tabular form or approximated by a simple function approximation algorithm. Monte Carlo (MC), Q-leaning (QL), State-action-reward-state-action (SARSA), policy gradient (PG), and actor-critic (AC) are examples of TRL approaches. For TRL-based BAMS applications, tabular QL was used for occupancy prediction and HVAC control to optimize the occupant comfort and energy consumption in Barrett and Linder (2015), and controlling the HVAC system and windows for mechanical and natural ventilation in Chen et al. (2018).

##### R2. DRL models

Recently, RL has taken advantage of the DL technology to reach phenomenal results. Typically, DL has been combined with RL due to its ability to capture all the intricate details of the knowledge and also perform complicated learning tasks that RL failed in doing so. This has given rise to DRL. In BAMSs and many other research fields, DRL is becoming a significant focus of scientists. The commonly used DRL methods are deep Q-learning (DQL), asynchronous advantage actor-critic (A3C), deep deterministic policy gradient (DDPG), and proximal policy optimization (PPO). A review of DRL applications for intelligent buildings energy management was presented in Yu et al. DQL was used for indoor and domestic hot water temperature control in Lissa et al. (2021) to optimize the home energy management system. In Wang et al. (2017), a DRL-based control system for office HVAC systems using an RNN-based actor-critic approach was presented. In Valladares et al. (2019), double Q-learning was utilized for energy optimization and thermal comfort control, while PPO method was applied in Azuatalam et al. (2020); Chemingui et al. (2020) for controlling the building’s HVAC systems for energy and thermal comfort optimization.

AL in all, RL models (TRL and DRL) are utilized for solving very complex problems that can not be fixed using traditional ML or DL models. They can also correct the errors occurring during the training stage. However, exceeding the number of required RL stages can result in an overload of states, and hence reducing the performance of RL models (Ding et al. 2019).

#### Ensemble methods (E)

Ensemble methods are a class of ML that deploy different aggregation strategies for combining multiple learning models and then achieving better predictive performance compared to the use of a unique learning algorithm.

##### E1. Boosting

It implies the gradual development of an ensemble learning using a set of ML models, where every new model occurrence is trained for emphasizing the training occurrences that previous models misclassified. In some applications, boosting can achieve better performance than bagging; however, it often looks after overfitting the training data. Random forest (RF), adaptive Boosting (Adaboost), and eXtreme gradient boosting (XGBoost) were among the most used boosting models for different AI-big data analytics tasks, such as overall building energy consumption forecasting (Zekić-Sušac et al. 2021; Xiao et al. 2021; Ferdoush et al.; Wang and Chen 2021; Yucong and Bo 2020), heating and ventilation load prediction (Sun et al. 2020), HVAC optimization (Li 2020), Space cooling load forecasting (Feng et al. 2021), load disaggregation and monitoring (Xiao et al. 2021), water monitoring (Somontina et al. 2018; Movahedi and Derrible; Golabi et al. 2020), IEQ monitoring (Mo et al. 2019).

Following, other variants have been then introduced and utilized for performing energy forecasting and load monitoring, IEQ monitoring, water management and occupancy detection in different kinds of buildings, including gradient boosting machine (GBM) (Gong et al. 2020), extreme gradient boosting machine (XGBM) (Gong et al. 2020), gradient boosting regression tree (GBRT) (Nie et al. 2021), LightGBM (Park et al. 2021; Wang et al. 2020).

##### E2. Bootstrap aggregating

It is also abbreviated as bagging and refers to the design of a new ML model by aggregating multiple models that have equal weights in the ensemble vote. Every model is trained using a randomly drawn subclass of training data for promoting the model’s variance. Various bagging models have been developed, modified and used to perform different tasks in BAMSs. For instance, bagging ARIMA (BARIMA) in de Oliveira and Oliveira (2018) is proposed to conduct a mid-long term load forecasting, while in Khwaja et al. (2015), a bagging neural network (BNN) is developed where the bagging concept is combined with neural networks (NNs) to improve short-term energy prediction. Moving on, in Hu et al. (2020), an enhanced bagged echo state network (BESN) is introduced to forecast energy. In Choi and Hur (2020), a bagging model is developed by setting RF, XGBoost and LightGBMs as the base learners. In Dehalwar et al. (2016), the authors introduce a bagged regression tree (BRT) that has been used for energy forecasting.

Moreover, bagging models have also been employed for water management in buildings using ensemble bagging tree (EBT) (Hasanzadeh Nafari et al. 2016), and thermal evaluation using bagged tree (BT) (Ahmad and Chen 2018), and fault detection using bagged auto-associative kernel regression (BAAKR) (Yu et al. 2017).

Overall, ensemble methods have been used in BAMSs since they can result in better predictive accuracy than individual models in complex systems/models Moreover, they are appropriate for scenarios with linear and non-linear data variables. However, ensembling is less interpretable, and the outputs of ensemble models are complex to explain and predict in most applications. In addition, a wrong selection of the models to be aggregated will arise lower predictive accuracy than individual models. Furthermore, ensemble models are generally computationally expensive and require much storage memory.

### Building environments and their characteristics

Buildings range in size, function, construction, design, and other attributes. Additionally, they present varying levels of potential hazards and risks to the occupants and the surrounding environment. However, buildings are primarily classified based on the utilization purpose that governs occupancy profile, sophistication level, and building design requirements. Building environments are further described in the following subsections.

#### Residential buildings

Residential buildings are mainly for private occupancy, designed and built for individuals or groups, providing the necessary facilities and utilities to satisfy living requirements. Spaces in residential buildings involve several activities, including sleeping, sitting, conveniences, cooking, dining, and others. Those functions can be in shared spaces or have exclusive rooms per function. They exist in various sizes and have different occupancy rates. A low occupancy density generally characterizes them. Examples of residential buildings are story houses, apartments, terraces, and condominiums. In addition to air conditioning and ventilation systems, lighting, and media equipment, several major appliances are regularly used in residential buildings, such as dishwashers, washers, dryers, refrigerators, freezers, stoves, water heaters, trash compactors, ovens, and others (Estiri 2014).

Residential buildings are typically equipped with simple BAMSs that provide the basic requirements of building management for inhabitants’ well-being and comfort. Standard manual control is used for the most part of their BAMSs. For instance, the decentralized control of the indoor environment is driven by the thermal comfort levels of the occupants. Thermal comfort is subjective to outside weather conditions that determine the indoor environment conditioning requirements, which are heating or cooling, humidification or dehumidification, and air ventilation (Do and Cetin 2018).

#### Office buildings

Office buildings are where people perform routine tasks, execute assignments and jobs for their employers, or provide passive or active, free of charge or remunerated services to the public. Types of workplaces vary in the form and requirements of the work and the variety of tools involved. Hence, they differ in size and the extent of personnel involvement and expertise. Familiar workplaces are office buildings such as law and corporate firms, commercial companies, post offices, banks, courtrooms, and similar places where people are involved in lengthy desk jobs or light-weight activities. Most of the spaces are offices, meeting rooms, or auditoriums of defined capacities. Additionally, they have shared areas such as corridors and lobbies. Office buildings require flexible and technologically-advanced working environments that are safe, healthy, pleasant, durable, and accessible towards promoting the users’ comfort, productivity, and working efficiency (Tanabe et al. 2013). It includes the accessibility to natural ventilation and natural lighting sources, and the availability of IEQ control and monitoring. The provision of localized indoor environment control allows users to adjust the air temperature, air movement, and other relevant indoor environment properties according to their preferences. They are characterized by their moderate operation schedules, and fairly regular and established user profiles. Additionally, some workspaces may involve many service recipients (Alsalemi et al.).

#### Healthcare centers

The indoor environment in healthcare centers is critical for the health, well-being, safety, and comfort of patients, visitors, and the staff, as well as for the medical utilities and services. It has to comply with specific standards related to temperature, infection, and odor control (Salonen et al. 2013). It plays a significant role in the quality of the provided medical service in terms of the treatment, healing, recovery processes, and the success of the conducted operations and procedures.

The various spaces in healthcare centers have different temperature regulation requirements. For instance, the success of surgical procedures depends in part upon the cold indoor conditions of the operating room to avoid the risks of anesthetic explosions, promote the comfort, productivity, and efficiency of the staff, and conserve the patient’s resources (Ellis 1963). On the other hand, burn units are regulated at temperatures between 28 and 33 degrees because burn injuries restrict the ability of patients’ bodies to stay warm (Fernández and Pablo 2021). Moreover, healthcare centers require a clean and sterile environment. Hospital-acquired infections are a major threat in healthcare centers (Lobdell et al. 2012). Hence, air ventilation and infection control are essential to control the potential contaminants and other suspended microorganisms, consequently lowering airborne disease risk. Additionally, air ventilation helps dispel odors, which improves the indoor conditions for the patients, staff, and visitors. Moreover, medical waste disposal and management is an essential aspect of the operation of healthcare centers as they are considered one of the main sites for the generation of hazardous waste (Aljabre 2002). The proper management of medical waste is essential to avoid health and environmental risks. Healthcare centers have protocols for the disposal of the generated waste according to their location. Additionally, healthcare centers are obliged to provide adequate security implementations for (i) the safety of patients, the public, and staff, (ii) the privacy and integrity of the patients’ data, (iii) the prevention of breaches against the BAMS, (iv) the management of the utilities and equipment, and (v) the prevention of injuries and unwanted occurrences.

#### Sports facilities

Sports facilities involve areas where individuals or groups engage in physical exercise, participate in athletic competitions, or attend sporting events. Examples of sports facilities are gymnasiums, cultural centers, stadiums, swimming pools, indoor and outdoor tennis courts, squash courts, training halls, and sports arenas. They encompass large and various spaces involving different types of activities. Sports facilities have distinct requirements for air conditioning and ventilation, thermal comfort, and lighting with unique usage and occupancy patterns. They are governed by the type of sports activity, the operating time, the season, and the geographical location of the facility (Trianti-Stourna et al. 1998).

Sports facilities are characterized by the variety of their architectural sophistication and sizes, deployed technologies, and their distinctive energy demand profile compared to other types of buildings (Elnour et al. 2022). For instance, stadiums are the most sophisticated ones, which occupy vast land space. Even though they are often infrequently used, their operation and running costs during a single event are substantial (Aquino and Nawari 2015). Aquatic centers are the second most popular sports facilities that host different water events and tournaments. They encompass other spaces such as changing rooms, shower rooms, and storage rooms.

Sustainability measures and implementation are deployed in sports facilities’ design, construction, and operations. They require extensive lighting, air conditioning, broadcasting, surveillance, and security requirements when operated to achieve successful sports events. The proper lighting in the sports facilities ensures good visual conditions. The event’s prosperous broadcasting is essential to delivering an entertaining, thrilling, and engaging experience for the athletes and fans. Given the considerable volume of user flow in sports facilities, emergency evacuation planning, users’ entry and exit management, security screening, and preventive measures are among the top priorities in sports facilities management (Hall et al. 2011). Additionally, sports facilities involve extensive body workouts and activity by the users, during which excessive heat and CO2 discharge occur. They demand mainly air conditioning and ventilation, especially for indoor sports events as well as water heating for pools and domestic use to maintain the comfort, health, and well-being of the users. Moreover, sports facilities require constant maintenance, servicing, and overseeing even when not used, such as grass fields, pools, water treatment, and sports equipment.

#### Commercial buildings

Commercial buildings have at least 50% of their floor spaces for commercial activities (Kiliccote and Piette), such as malls, retail, and food services. Malls and restaurants are typical commercial buildings of various sizes and complexity. They include shops, cafes, kitchens with several commercial appliances, storage rooms, pantries, a refrigerated space, offices, dining areas, and public restrooms. They demand maintaining a clean and well-conditioned environment. For example, in restaurants and coffee shops, compliance with proper food storage and preparation standards is required to reduce the risk of spoiling food and eliminate the risk of incidents jeopardizing the well-being of the users as well as the reputation of the restaurants (El-Sharkawy and Javed 2018).

Air ventilation affects the health and safety of workers and customers and can influence food sanitation levels. The chiefs and the kitchen staff in restaurants are exposed to air pollutants generated from cooking for long periods. Hence, they may suffer potential respiratory and cardiovascular problems in the long run (Juntarawijit and Juntarawijit 2017). Also, they are subjected to high levels of heat generated from cooking activities, decreasing the staff’s productivity. Additionally, excessive unpleasant odors or poor air conditioning in restaurants can result in an unpleasant experience for the customers. In addition, malls and shopping centers are commercial buildings where goods or services are sold to customers. They may include ample parking spaces, escalators, elevators, and various outlets such as department stores, food courts, amusement and theme parks, and movie theaters. Safe and comfortable indoor conditions are essential to provide a convenient and enjoyable experience for users and maintain a flourishing business with efficient energy consumption to contain the incurred running and operating costs.

Commercial buildings are famous for their exceptional operating schedule and occupancy patterns. They run for about more than 12 hours all week, and they have peak occupancy during weekends and significant volumes of user flow. They utilize extensive closed-circuit television (CCTV) surveillance, lighting, and air ventilation and conditioning systems. Additionally, fire prevention, suspension, and other security and alarm systems are crucial elements of their management systems to ensure dependable and safe circumstances for the users. Overly, the proper management of commercial buildings is essential to maintain a lucrative operation.

#### Industrial buildings

Industrial buildings include buildings used for the generation and distribution of power, manufacturing products such as food, apparel, electronics, petrochemicals, construction materials, automobiles, the processing of raw materials, and many others. They have minimal and relatively low user flow for security purposes, such that they are only accessible to individuals with privileges. However, they involve energy-intensive and delicate machinery. They are generally equipped with sophisticated BAMSs that support the security and the centralized control requirements. Industrial buildings are equipped with robotics, industrial devices, and software-defined production processes. They require a high level of automation, given the nature of the processes involved and the tasks performed. In addition, they may involve delicate processes that are associated with health, social, and environmental risks. Industrial sites and environments can result in air and water pollution due to the generated by-products and the released unwanted toxins of the occurring processes. Hazards from combustion and unstable reactions can lead to highly harmful accidents due to the sudden release of material at high temperatures or pressures (Englund 2007). Additionally, fire hazards are common in industrial facilities, which can endanger the lives of staff and can result in substantial economic losses and environmental implications. Industrial facilities must be safe, secure, and productive. Proper process control, air ventilation, treatment and conditioning, and waste management are crucial to managing the safety and health of the staff as well as the general public and the surrounding environment. Security is an essential dimension in the operation of the BAMSs of industrial buildings.

#### Academic buildings

Academic buildings are used to conduct teaching activities such as schools, academies, universities, colleges, technical institutes, etc. They encompass classrooms, lecture halls, libraries, student centers, dining halls, laboratories, computer labs, offices, and service areas necessary for the proper functioning of the academic programs. Individuals of various age groups are frequent users of educational facilities, and they engage in multiple types of activities. A convenient and safe environment in academic facilities is an essential requirement for the education process. It affects the well-being and comfort of students, faculty members, and other staff, hence their productivity and working efficiency. A comfortable and safe environment has been identified as an essential element for enhancing the learning of students (Muhammad et al. 2014). Over-heated and poorly ventilated classrooms can result in the discomfort of students and educators, and consequently diverts their attention and affects their abilities to concentrate (Roelofsen). Adequate lighting in the facilities of academic buildings is vital to the comfort and well-being of the students to create an attractive and engaging learning environment and avoid eye strain. Additionally, students’ health and well-being are essential for their learning process. The indoor environment influences students’ attendance and hence their study. Students need to be in good health to be able to study well. Therefore, spaces in academic buildings should be well conditioned and ventilated to avoid altering users’ well-being, spreading airborne diseases spread, and disrupting students’ learning. Lastly, a brief summary is presented in Table 2 to compare the characteristics of the different buildings discussed above.

**Table 2:** A comparison between the different types of buildings

Building type	BAMS type	Use	Spaces type	Activity type	Size	Occupancy/Usage	Characteristics	Requirements	Example
Residential	Simple for basic requirements for inhabitants’ well-being and comfort, standard manual control	Dwelling	Spaces for sleeping, sitting, conveniences, cooking, dining	- Dwelling, Light weight activities	Small to moderate	Low to moderate	- Light operations, and causal equipment/appliances - Consume relatively substantial amounts of energy and accompanied by peak demand issues	Iinhabitants’ well-being and comfort	Houses, apartmentscondominiums
Office	Standard for natural ventilation, natural lighting, local indoor environment control, surveillance and safety system	Business	Offices, meeting rooms, auditoriums, corridors, lobbies	- Routine tasks, services to public - Desk jobs or light-weight activities	Small to moderate	Moderate to high	- Fairly regular and established user profiles	healthy, flexible, durable, productive, efficient, and comfortable working environment	Firms, banks,post offices
Heathcare	Advanced for air treatment, ventilation, and temperature regulation, security, surveillance, servicesequipment management	Health services	Treatment rooms, care units, examination rooms, laboratories, blood banks, operating rooms, patients rooms, waiting rooms, corridors, lobbies	- Delicate medical procedures - Lab experiments	Average to large	Moderate to high	- Year-round operation, frequent and considerable users flow- include sophisticated and expensive equipment for diagnostic and treatment- associated with health and environmental risks.	-Health, well-being, safety, and comfort of patients, visitors, the staff, medical utilities and services-comply with specific standards related to temperature, infection, and odor control. - Clean and sterile environment	Hospitals, clinicshealth centers
Sports	Advanced system for extensive lighting, air conditioning, broadcasting,surveillance, and security	Sporting events and activities	Training rooms, spectators areas, changing rooms, shower rooms, storage rooms, offices, lobbies, corridors	- Sporting activities of different levels and types	Average to large	Moderate to high	-High seasonal usage patterns of high users flow, -Encompass various space types, - Substantial running and operation costs	-Distinct requirements for air conditioning and ventilation, thermal comfort - Specific visual conditions and broadcasting requirements	Stadiums, sports centers,swimming pools
Commercial	Inclusive system for closed-circuit television, surveillance, lighting, air ventilation and conditioning	Commerce	Shops, cafes, dining areas, kitchens, storage rooms, offices, lobbies, corridors	- Standard, lightweight activities	Average to large	Moderate to high	Year-round operation with frequent peak periods of considerable users flow	-Clean and well-conditioned environment- Proper air ventilation for maintaining health and safety of workers and customers	Shopping malls, grocery stores,restaurants
Industrial	Sophisticated system for centralized automation and management	Industries	Offices, control rooms, computer rooms, machinery and process rooms, electrical and chemical plants	- Light to heavy weight activities	Average to large	Low to moderate	Involve limited and consistent users flow, energy-intensive and delicate machinery and processes associated with health, social, and environmental risks	- High level of automation - safe, secure, and productive operation - waste management	Factories, power stations
Academic	Inclusive system for surveillance, lighting, air ventilation and conditioning, and security	Education	Classrooms, offices, meeting rooms, dining halls, laboratories, computer labs, lobbies, corridors	- Light to heavy weight activities	Average to large	Moderate to high	- Seasonal usage patterns of moderate to high flow	- A convenient, comfortable and safe environment for students and staff - Adequate lighting	Universities, colleges,schools

### Computing platforms

#### Cloud computing

The advancement of cloud computing platforms has opened new opportunities for BAMSs to take control of operations on a large scale. Thus, BAMSs that consist of networked sensors and actuators, have been recently adapted to be able to connect to different cloud-based services (Alsalemi et al. 2020). The latter can provide data storage, connectivity, and powerful computing resources. To that end, significant efforts have been devoted to developing cloud-based big data analytics solutions in BAMSs (Bode et al. 2019). For instance, a voice-activated system for remotely monitoring BAMSs using cloud computing is presented in Valenzuela et al. (2013). While in Khattak et al. (2019), the idea of developing vehicular clouds for smart buildings and smart city applications is investigated. Moving on, in Stergiou et al. (2018), the security and privacy concerns along with the efficiency of cloud platforms are analyzed. In Delsing (2017), local cloud IoT automation is studied to promote the use of distributed IoT automation solutions.

Despite the significant effort made during the last decade to promote the use of cloud-services to run BAMSs, some drawbacks are still causing issues to users and operators, among them (i) the increased cost and communication overheads, (ii) the privacy and security concerns, especially when private data is transmitted to a centralized server for processing (Mohamed et al. 2018).

#### Edge computing

Edge computing it refers to performing data pre-processing, data fusion for different sources and AI-big data analytics at the edge of the network i.e. sensor nodes (Ray et al. 2019). Also, it enables optimizing cloud computing platforms due to its capability to use the processing power of IoT devices for filtering, pre-processing, aggregating and storing IoT sensor data. These tasks can correspondingly be conducted in real-time using convenient analytical tools (Sharma et al. 2018), while cloud platforms perform further enrichment, aggregation and running complex analytics on the filtered data. To that end, the new advances in BAMSs combined with the latest generation of IoT devices make it possible to bring the intelligence and computing tasks to the edge nodes in close proximity to the building’s IoT devices (Zakharchenko and Stepanets 2019; Khan et al. 2020). Moreover, a new generation of open software platforms hosted on edge nodes are enabling access to the building data and advanced AI-big analytics deployed on these platforms are providing the technology to create value from this data by transforming data from building environments into actionable information. Various open edge platforms have recently been proposed, e.g. IOTech’s Edge Xpert,^3^ Echelon SmartServer IoT platform,^4^ JENEsys Edge,^5^ etc.

#### Fog computing

Fog computing represents a decentralized computing strategy where data storage, data processing and computing resources are located in the middle layer situated between edge devices and cloud. Typically, IoT smart sensors and submeters periodically collect the data and forward it to a gateway that acts as a fog device (Javadzadeh and Rahmani 2020). In this line, BAMSs can benefit from streaming data over a layer of fog devices (or nodes) to become more connected, where data can be analyzed to detect abnormalities for example, and autonomously react, if authorized, for compensating the problems or fixing the issues. Otherwise, fog nodes will send the convenient requests to the cloud (or services higher up the fog hierarchy) for making further skilled and powerful technical analysis using complex ML models (Ferrández-Pastor et al. 2018; Aazam et al. 2018).

For instance, in some situations that require real-time decision-making, e.g. shut down appliances or equipment before being damaged or adjust crucial process parameters, edge devices or fog nodes can rapidly act with millisecond-level latency, while it is not possible to reach real-time decision making using cloud data centers (Rocha Filho et al. 2018). Therefore, the use of fog computing or edge computing helps avoid potential latency problems, delays an/or network/server down-times that can lead to different kinds of accidents or reduced service optimization and efficiency (Maatoug et al. 2019).

#### Hybrid computing

Hybrid computing refers to the case when the aforementioned computing architectures, i.e. edge computing, fog computing and cloud computing, are used together to process and analyze data (Himeur et al. 2021; Zhang et al. 2020). In this context, based on the application scenario and computation requirement, some data processing tasks could be made at the edge devices and/or fog nodes, while high-level data processing tasks (e.g. feature extraction, classification, anomaly detection, etc.) could be performed at the cloud data centers (Himeur et al. 2020).

### Applications

#### Facility and asset management

Facility management to eliminate waste is among the benefits of using AI-big data analytics in BAMSs and can perform in diverse forms. For instance, using an AI strategy, a bathroom supplies monitoring company has saved up to 40% in of the total cost by installing a sensors that collect and send information about the utilization levels of toilet paper rolls and soaps (Gaboalapswe 2019; Sayed et al. 2022). Similar techniques are also be deployed for monitoring sports facilities, commercial buildings, office supplies, and other building necessities (Himeur et al. 2021; Idowu et al. 2016).

For instance, in sports facilities there is an emergency to improve the BAMS services to meet consumer’s growing experience needs, and hence, overcome various issues, e.g. poor resource sharing, weak flexibility of response and slow transmission of information, and instability of aero-thermal comfort, which are considerable affecting the end-users’ experience and restricting the development of sport venues (Zhong et al. 2020). To that end, a great attention has been put recently to design intelligent BAMS architectures of sport centers. This helps in interconnecting multiple subsystems, improving the interoperability, integrating information, realizing the integration of data application network, and achieves the goal of resource sharing and function upgrading. In Xiao-wei (2020), an AI-big data analytics platform is built using SVM-back propagation neural network (SVM-BPNN) for (i) predicting the end-user flow in the sport facility, (ii) providing recommendations to adjust the service plan, and (iii) improving the overall management and the end-users’ experience. Moving on, in Wan et al. (2021), as the cyber-security is a challenging issue in sports facilities due to the number of spectators and players and the large number of sport events organized, an AI-assisted cyber-physical system (AI-CPS) is integrated to the BAMS for promoting network security and predicting cyber attacks and adversaries.

On the other hand, because developing an appropriate setpoint temperature for the HVAC system is a crucial challenge, the authors in Aparicio-Ruiz et al. (2021) identify such temperature using a KNN-based dynamic adaptive comfort technique. It relies on the idea that occupants’ thermal comfort in a building has different acceptability levels, which can be used for learning the comfort temperature corresponding to the average running temperature. Thus, this helps define the adequate range of indoor temperature. While in Carreira et al. (2018), Carreira et al. introduce a framework for tracking building end-users’ group preferences, learning from them, and automatically managing HVAC systems. This framework is built by tracking building users using an RFID card, interacting with them on a mobile app, computing setpoints, and sending instructions to the HVAC sub-system over a gateway. Additionally, a K-means algorithm has been used for configuring the setpoint, in line with a prediction based on the current building status.

#### Load forecasting

In BAMSs, forecasting energy consumption is of significant importance to enable an effective management of energy, in which AI-big data analytics techniques play an essential role. In doing so, load patterns (and ambient conditions) are constantly collected from diverse building smart-meters and then fed into the AI models to predict energy usage. Because of the real-time characteristic of short-term forecasting, it has been more challenging than generic forecasting. Thus, various AI-big data analytics models have been proposed (Chou and Tran 2018; Ahmad and Chen 2018; Seyedzadeh et al. 2018; Fathi et al. 2020). In Pham et al. (2020), a random forests (RF) model is introduced to perform a short-term energy load prediction at an hourly sampling rate in various buildings by using different energy consumption datasets. In Seyedzadeh et al. (2019), the authors investigate the performance of diverse popular ML algorithms to predict buildings heating and cooling energy usage. Accordingly, specific tuning has been carried out for every ML algorithm using two building energy consumption datasets generated in EnergyPlus and Ecotect. In Ribeiro et al. (2018), a transfer learning based load prediction scheme is introduced, where energy consumption data of different buildings are used to forecast the load of a new building. This approach can work with various ML algorithms with pre- and post-processing phases.

In Moon et al. (2018), Moon et al. propose an energy prediction model using diverse ML models, including ANN, SVR, and PCA-factor analysis (PCA-FA). Data from four buildings in an academic institution have been used for evaluating the performance of these models. In Ahmad et al. (2020), an intelligent load prediction scheme is proposed using generated sampled data-based Gaussian process regression model (GSD-GPRM), regression binary decision tree (RBDT), bootstrap bagging of regression trees (BBRT) and binary multiclass classification decision tree (BMCDT). In Idowu et al. (2016), supervised ML algorithms are used to develop a load forecasting model using SVM, regression tree, feed-forward neural network (FFNN), and multiple linear regression (MLR). Moving on, in Ahmad et al. (2018), diverse supervised ML models are implemented to predict energy consumption at short, medium, and long-term levels in different building environments, namely compact regression Gaussian process (CRGP), binary decision tree (BDT), generalized linear regression model (GLRM) and stepwise Gaussian processes regression (SGPR). In Chou and Ngo (2016), a short-term based energy prediction system is proposed using a seasonal autoregressive integrated moving average (SARIMA) model along with a metaheuristic firefly algorithm-based least squares support vector regression (MetaFA-LSSVR) model. Typically, this framework uses (i) the SARIMA architecture for linearing energy observations, and (ii) the MetaFA-LSSVR model for capturing nonlinear energy patterns.

**Fig. 4:**
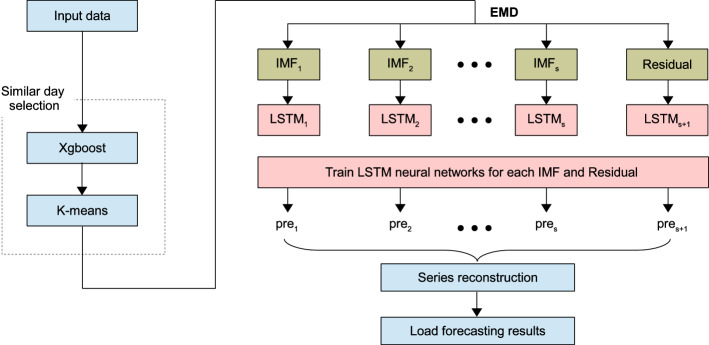
Flowchart of an energy forecasting system based on AI-big data analytics (Zheng et al. 2017)

In Li et al. (2021), a transfer-learning-based ANN scheme is developed to predict short-term energy consumption in information-poor buildings. The efficiency of transfer learning in improving the prediction accuracy has been demonstrated using limited training data. Moving on, in Grolinger et al. (2016), a short-term energy consumption prediction of sports facilities, which is considered as a challenging scenario due to the variations caused by by the hosted events, is performed using NN and SVR. In Zheng et al. (2017), a short-term energy prediction approach using an empirical mode decomposition (EMD)-LSTM-based RNN is proposed with a Xgboost model to select feature patterns based on a feature importance evaluation. In a similar manner, in Haq et al. (2021), a sequential learning-based load forecasting algorithm is developed and used in both residential and commercial buildings. Accordingly, this framework implements a convLSTM integrated with BiLSTM (ConvLSTM-BiLSTM) and compares its performance with various sequential models, including ConvLSTM integrated with BiLSTM, LSTM, auto-encoder (AE), multi-layer Bi-LSTM (MBiLSTM), BiLSTM-AE, GRU, and CNN with multilayer bidirectional GRU (CNN-MB-GRU). Fig. 4 illustrates a flowchart of an energy forecasting system based on AI-big data analytics. Specifically, a short-term energy consumption prediction is performed using EMD-LSTM neural networks with a Xgboost algorithm to extract importance features. Besides, Moradzadeh et al. (2020) propose a heating and cooling load forecasting scheme that is based on MLP and SVR in residential buildings. These models help identify a linear mapping between inputs and outputs. MLP has outperformed SVR in terms of the recall metric, where a recall of 99.93% has been achieved. To summarize, Table 3 compares various AI-Big data analytics frameworks used for energy forecasting, in terms of AI model, forecast horizon, building environment, year of appearance, method description and evaluation metrics.

**Table 3:** A summary of AI-Big data analytics frameworks proposed for energy forecasting in buildings

Ref.	AI model	Forecasthorizon	Building nature	Year	Description	Evaluation metrics			
						RMSE	MAE	MAPE	Others
Skomski et al. (2020)	seq2seq	Short-term	Office	2020	Demonstrate the efficiency of seq2seq RNNs for load prediction using a restricted feature set	✓			nRMSE
Bessani et al. (2020)	Bayesian networks	Short-term	Residential	2020	Handle the volatility and the uncertainty of buildings’ loads	✓		✓	nRMSE, MedAE
Ribeiro et al. (2018)	transfer—based MLP, SVR	Long-term	Residential	2020	TL-based trend and seasonal adjustments to predict cross-building load		✓		MSE
Ahmad et al. (2020)	GSD-GPRM, RBDT, BBRT, BMCDT	Short-, long-term	Office	2020	Building load prediction in non-climate sensitive and climate-sensitiveconditions		✓	✓	CV
Moon et al. (2018)	ANN, SVR, PCA-FA	Short-term	Academic	2020	Energy prediction of higher educational institutions	✓	✓	✓	
Zhang et al. (2020)	LSTM, GRU, CIFG	Short-term	Public	2020	Hybrid DL-based energy prediction combined with an interpretation process	✓	✓		CV-RMSE, $R^{2}$
Wen et al. (2020)	RNN-GRU	Short-, mid-term	Residential	2020	Achieve well performance with limited input variables	✓	✓	✓	
Park et al. (2020)	XGBoost, RF, DNN	Short-term	Industrial	2020	A Two-Stage energy consumption prediction			✓	CVRMSE
Khamma et al. (2020)	GAMs	Short-term	Office	2020	Embed domain knowledge and prior understanding of buildings into the prediction model				CVRMSE, NMBE
Somu et al. (2020)	ISCOA-LSTM	Short- , mid-, long-term	Residential	2020	Accurate and reliabale data driven load forecasting	✓	✓	✓	MSE,Theil U1, U2
Liu et al. (2020)	A3C, DDPG, RDPG	Mid-, long-term	N/A	2020	Improve the forecasting accuracy with increasing computation time	✓	✓		$R^{2}$, CV
Zhang et al. (2020)	DBN-DEEM	Short-term	Residential	2020	Predict stochastic energy consumption using Cyclic feature (CF) extracted via spectrum analysis	✓	✓	✓	*r*
Lu et al. (2020)	CEEMDAN-XGBoost	Short-term	Intake towers	2020	Have half of prediction error of XGBoost using real-world data for a period of 8 years				RMSPE, Theil U1, U2
Wang et al. (2020)	stacking model	Short-term	Academic	2020	Building load forecasting using model integration	✓	✓	✓	CVRMSE
Somu et al. (2021)	$\kappa$CNN-LSTM	Long-term	Academic	2021	Capture the load spatio-temporal features and aid in decision making	✓	✓	✓	MSE
Yuan et al. (2020)	WNN-cuckoo search	Mid-term	Commercial	2020	Optimally tuning the WNN parameters CS with				DMAPE, AE
				2020	a real-world validation				
Mawson and Hughes (2020)	DFNN, RNN	Mid-term	Industrial	2020	Load forecasting and condition monitoring in manufacturing buildings	✓	✓		
Bui et al. (2021)	LSTM	Long-, and short-term	Residential	2021	Multi-behavior with bottleneck features LSTM for to predict energy consumption	✓	✓	✓	NRMSE
Dun and Wu (2020)	Grey model	Long-term	Residential	2020	Load forecasting of three kinds of buildings, i.e. rural, public and urban buildings			✓	
Khan et al. (2021)	LSTM-KF	Short-term	Residential	2021	Learning to statistical model for ensemble predicting of energy consumption	✓	✓	✓	
Li et al. (2021)	TL-based ANN	Short-term	Residential		Load prediction of information-poor buildings			✓	NTR
Grolinger et al. (2016)	NN-SVR	Short-term	Sport-venues	2016	Load forecasting in a challenging scenario with high variations caused by the hosted events			✓	
Pinto et al. (2021)	RF, GBR	Short-term	Office	2021	Combine multiple learners to optimize the learning process			✓	

#### Energy efficiency

One area that can get immensely benefited from AI-big data analytics is energy efficiency in buildings. This is because of the way a building consumes energy can be quite variable, and is related to various parameters, e.g. the nature of the building, the energy provider, the sources of the energy, the number of devices, the number of end-users/occupants in any building, the behavior of end-users/occupants, etc. Himeur et al. (2021); Fatema et al. (2020). Moreover, comprehending the energy consumption habits of a building is the first and most critical step to achieve energy efficiency. This way, AI-big data analytics and energy efficiency can go hand in hand towards the goal of optimizing energy consumption and reducing the amount of wasted energy without compromising the comfort level of end-users and the level of efficiency and productivity in a company or industry Sardianos et al. (2021).

In (Yu and Chiller), Yu et al. propose an open IoT cloud-based ML system, namely AI Chiller, to promote energy efficiency in buildings by optimizing the consumption of the HVAC system. An AI-big data analytics scheme based on an RNN-LSTM architecture and to analyzing and fusing BAMS environmental footprints has been developed and combined with a genetic algorithm to achieve 10% savings. In Al-Ali et al. (2017), energy saving in residential buildings in the Gulf region is achieved using IoT, off-the-shelf business intelligence, and big data analytics platforms.

#### Predictive control and thermal comfort

One solution to save buildings’ energy is using model predictive control (MPC). It aims at developing predictive models for (i) simulating input-output interactions; and (ii) helping users to identify optimum control actions that drive the predicted outputs to the desired references. In this context, ML models predict energy demand and simulate MPC control techniques to save energy and optimize end-users’ comfort. Typically, these models can provide decision bases for selecting optimal MPC control actions Serale et al. (2018); Mariano-Hernández et al. (2021). In Gao et al. (2020), building thermal comfort control is conducted using RL, where a deep feed-forward neural network (FNN)-based method is introduced. The latter helps predict consumers’ thermal comfort before introducing a deep deterministic policy gradients (DDPGs)-based scheme to optimize thermal comfort. In Yang et al. (2020), an MPC approach based on an RNN with nonlinear autoregressive exogenous (NARX) architecture, namely NARX-RNN, is proposed to optimize air conditioning and mechanical ventilation (ACMV) in a hospital office and hence save energy and optimize thermal comfort. Similarly, in Yang et al. (2021), the same methodology is experimentally implemented to control the ACMV systems in office and lecture theatre (LT) testbeds in real-time. In Chen et al. (2020), Chen et al. developed an MPC approach based on deep transfer learning to optimize the HVAC operation in smart buildings.

Moving forward, in Bünning et al. (2020), an RF-based data predictive control (DPC) scheme is proposed using convex optimization and affine functions. This help in controlling energy consumption and temperature in a room of a real-life apartment. In Yang and Wan (2022), Yang et al., RNN-NARX-based MPC is introduced with instantaneous linearization for ACMV optimization. Table 4 summarizes some of the recent ML-based MPC frameworks described above, their characteristics, and their contributions. Most concentrate on controlling HVAC and ACMV systems in buildings since they consume the most significant proportion of building energy. Thus, this significantly impacts the thermal comfort of buildings’ occupants.

**Table 4:** A summary of MPC-based frameworks, their characteristics and contributions in saving energy and optimizing thermal comfort

Work	ML model	Building type	System	Best performance	Control objective
Gao et al. (2020)	FNN + DDPGs	Public buildings	HVAC	4.31%HVAC energy saving	Save energy and improve thermal comfort.
Yang et al. (2020)	ANN-NARX	Office and lecture theater	ACMV	58.5% cooling thermal saving	Energy saving and thermal comfort optimization.
Yang et al. (2021)	RNN-NARX	Office and a lecture theater	ACMV	52% reduction of cooling energy	Save energy and optimize thermal comfort. in experimental testbeds
Chen et al. (2020)	MLP-based transfer learning	Residential buildings	HVAC	MSE=0.16	Optimize energy efficiency and thermal comfort.
Bünning et al. (2020)	RF	Residential buildings	HVAC	24.9% of cooling energy saving	Optimize energy consumption withoutcompromising thermal comfort
Yang and Wan (2022)	RNN-NARX	Office in a hospital	ACMV	26–31.6% cooling energy savings	Save energy and optimize thermal comfort
Li and Tong (2021)	Encoder-decoder RNN	Residential/public buildings	HVAC	4–7% energy saving	Energy saving and smart control of thermal environment
Mtibaa et al. (2021)	CAM- LSTM	Multi-zone buildings	HVAC	MAPE = 0.0872%	Save energy, predict peak power and improve thermal comfort

#### Anomaly and fault detection and diagnosis

Failures in building electric networks and devices’ operation cycles may result in excessive energy losses and extra costs. To alleviate these issues, AI-big data analytics are a prevalent tool that enables detecting faults and disturbances early enough and predicting maintenance. That is possible by implementing continuous energy consumption monitoring to create ”an early warning system” empowered with AI-big data analytics strategies and pattern recognition models to notify the end-users and operators Alsalemi et al. (2020). Accordingly, information related to energy consumption, environmental conditions, and occupancy patterns is fed into the AI black-boxes to identify and classify deviations. Once deviations are classified, their causes are determined before taking appropriate measures for their prevention Himeur et al. (2020), Alsalemi et al. (2020).

In this regard, a plethora of AI-based frameworks have been proposed to develop AI-big data analytics platforms that allow building energy efficiency Himeur et al. (2021). In Himeur et al. (2020), a DNN model and the micro-moment concept have been used to identify energy consumption deviations. A micro-moment rule-based algorithm is employed to extract load features of daily intent-driven energy usage moments. Next, DNN is applied to classify and determine abnormal consumption classes automatically and then compare the performance with various scenarios using conventional ML classifiers, e.g. LR, LDA, NB, SVM, RF, KNN, DT, ensemble classifier, and MLP. Similarly, in Himeur et al. (2021), unsupervised and supervised anomaly detection schemes are introduced to promote energy saving in academic and residential buildings. OCSVM is applied to extract abnormal energy consumption patterns from unlabeled data, while an improved kNN classifier is proposed to process annotated consumption footprints that are benchmarked using the micro-moment concept. In Xu and Chen (2020), a hybrid model using RNNs with quantile regression (QR) is proposed for anomaly detection in residential houses towards improving the performance of the building and reducing energy waste (Table 5).

**Table 5:** A summary of AI-Big data analytics models used for anomaly and fault detection and diagnosis

Ref.	AI model	Fault			System anomaly	Targeted system	Year	Description	Evaluation metrics			
		Sensor	Component	Actuator					ACC	MAE	MSE	Others
Himeur et al. (2020)	DNN				${\small \checkmark }$	Building appliances	2020	Classify and determine abnormal building energy consumption.	${\small \checkmark }$			Precision, F1-score, AUROC curves, confusion matrix
Himeur et al. (2021)	OCSVM, kNN				${\small \checkmark }$	Building appliances	2021	Classify and determine abnormal building energy consumption.	${\small \checkmark }$			F1-score
Xu and Chen (2020)	RNN, QR				${\small \checkmark }$	House	2020	Detect abnormal energy consumption.				PICP, PINAW
Taheri et al. (2021)	RNN	${\small \checkmark }$	${\small \checkmark }$	${\small \checkmark }$		HVAC systems	2021	Investigate various DRNN configurations to perform HVAC system fault diagnosis.	${\small \checkmark }$			Precision, recall, F1-score, cross entropy, confusion matrix
Yun et al. (2021)	NN-SAE		${\small \checkmark }$	${\small \checkmark }$		AHUs	2021	Detect and diagnose common AHUs faults to enable reliable maintenance.				Precision, recall, F1-score
Li et al. (2021)	GAN		${\small \checkmark }$			HVAC systems	2021	Detect and diagnose common HVAC system faults and addressing the issues of labeled data availability and data imbalance.	${\small \checkmark }$			FPR
Elnour et al. (2020)	AANN	${\small \checkmark }$				HVAC systems	2020	Perform sensor data validation and fault diagnosis of HVAC system sensor faults using semi-supervised learning.	${\small \checkmark }$			TPR, FPR, recovery rate, deviation rate, noise reduction rate
Elnour and Meskin	2D CNN			${\small \checkmark }$		HVAC systems	2021	Diagnose HVAC system single actuator faults using supervised learning.	${\small \checkmark }$			Precision, recall, F1-score, specificity
Dey et al. (2020)	MC-SVM		${\small \checkmark }$	${\small \checkmark }$		HVAC system	2020	Provide automated detection and diagnosis of equipment failures in HVAC systems’ terminal units.				Precision, recall
Bode et al. (2020)	LR, kNN, CART,RF, NB, SVM, NN		${\small \checkmark }$			Heat pumps	2020	Investigate fault detection approaches for operational heat pumps using machine learning algorithms.	${\small \checkmark }$			MCC
Shahnazari et al. (2019)	RNN	${\small \checkmark }$		${\small \checkmark }$		HVAC systems	2019	Develop models and a fault diagnosis methodology for HVAC systems.	${\small \checkmark }$	${\small \checkmark }$	${\small \checkmark }$	
Dey et al. (2020)	Clustering		${\small \checkmark }$	${\small \checkmark }$		FCUs	2020	Remote detection of fan coil units common faults.				Silhouette indexing, Davies-Bouldin, maximal gap
Han et al. (2019)	LS-SVM		${\small \checkmark }$	${\small \checkmark }$		Chiller system	2019	Diagnose common faults in centrifugal chillers.	${\small \checkmark }$			
Liu et al. (2021)	CNN		${\small \checkmark }$	${\small \checkmark }$		Chiller system	2021	Assess transfer learning for fault diagnosis methods in chillers.	${\small \checkmark }$			Recall, F1-score, precision confusion matrix
Zhu et al. (2021)	DANN		${\small \checkmark }$	${\small \checkmark }$		Chiller system	2021	Apply transfer learning for chiller fault diagnosis.	${\small \checkmark }$			Accuracy improvement degree
Han et al. (2020)	kNN, SVM, RF		${\small \checkmark }$	${\small \checkmark }$		Chiller system	2020	Assess using ensemble learning for chiller fault diagnosis.	${\small \checkmark }$			F1-score, confusion matrix
Choi and Yoon (2021)	NN-AE	${\small \checkmark }$		${\small \checkmark }$		BAMS	2021	Investigate variants of the AE-based fault diagnosis approach for building automation systems.	${\small \checkmark }$			Precision, F1-score

Additionally, fault detection and diagnosis in HVAC systems, being the most extensively operated equipment, has been covered widely in the literature. For instance, the utilization of the different configurations of deep RNNs is investigated in Taheri et al. (2021) to perform fault detection and diagnosis of common HVAC system faults, such as the malfunction and leakage of valves and dampers and sensor bias faults. A comparative study is presented comparing the performance of a DRNN-based diagnosis approach with RF and GB algorithms. In Yun et al. (2021), a neural network-based supervised auto-encoder (NN-SAE) - which is an auto-encoder with two outputs that are the classification label and the reconstructed signal- is proposed for air handling units fault detection and diagnosis before its validation using ASHRAE experimental data. The two outputs of the NN-SAE have been then processed to determine the diagnosis decision reliability. This approach has been compared with conventional ANN and SVM algorithms, and it has been found reliable as it considers undefined situations.

Additionally, a 2D CNN-based HVAC system actuator fault diagnosis is introduced in Elnour and Meskin, in which the system’s measurements and control signals were configured into multi-channel images and then processed using the 2D CNN-based diagnosis framework. CNNs are characterized by their high-performance accuracy and powerful capability in learning and realizing complex functions and interdependency from any given data. While in Liu et al. (2021), a CNN-based chiller fault diagnosis method is developed for building energy systems. Additionally, a TRL-based scheme is assessed to investigate the potential of using a pre-trained CNN-based fault diagnosis approach for chillers with different specifications, which is useful when available data is limited in size and/or types of operating conditions/faults captured.

In Dey et al. (2020), a big-data framework is presented to enable automated HVAC system fault diagnosis in large scale buildings in which a feature extraction approach is proposed to reduce the data dimensionality, and a multi-class SVM (MCSVM) algorithm is utilized to develop the diagnosis model. It aims to provide energy savings through preemptive maintenance, behavior analysis, and predictive building identification. In Bode et al. (2020), various ML algorithms are investigated to perform fault detection on a heat pump system, which are LR, kNN, classification and regression tree (CART), RF, NB, SVM, and NNs. This study demonstrates the effect of the data quality and amount and the limitations on the system’s features availability (i.e, types of available sensors) on the performance of the developed ML models. While in Han et al. (2020), a supervised hybrid fault diagnosis model using SVM, kNN, and RF is developed for chiller fault diagnosis such that the three models are developed independently to perform fault diagnosis; then the final decision is made based on the plurality voting method. It was found that ensemble learning contributes to diagnostic performance improvements. In Li et al. (2021), a fault diagnosis approach is proposed for the common component faults in the HVAC system of an office building using a modified GAN. The proposed approach enables leveraging the labeled and unlabeled data simultaneously such that it aims to process the unlabeled data and utilize the limited information from the labeled ones to conclude the diagnosis decision.

Those fault diagnosis approaches mainly require sufficient labeled data for training, which can be unavailable or complex, and costly to obtain. Therefore, several studies have developed unsupervised and semi-supervised diagnosis strategies as in Elnour et al. (2020), where an auto-associative neural network (AANN) is utilized for sensor data validation and fault diagnosis in HVAC systems using semi-supervised learning. It demonstrates a compelling performance in sensor error correction, data replacement of unavailable sensors, measurement noise reduction, and sensor inaccuracy correction. Also, it is effective for both single and multiple sensor faults diagnoses. In Zhu et al. (2021), transfer learning is applied to develop a chiller fault diagnosis approach that only requires the system’s normal operation data using domain adversarial neural network (DANN). While in Shahnazari et al. (2019), a distributed diagnosis approach using RNNs utilizing the normal system operation data is developed for multiple sensors and actuator faults diagnosis. It is based on developing intercommunicating fault detection and isolation (LFDI) agents for the various HVAC subsystems, i.e., cooling coil, VAV box, etc., and each LFDI agent is composed of two RNN-based models. It demonstrates promising capability in fault diagnosis. However, it is excessively computationally demanding, given the two RNN-based models included in each agent.

Additionally, a multi-level automatic fault detection framework is proposed in Dey et al. (2020) for fan coil units (FCUs). Feature extraction followed by data clustering are applied to identify faulty and healthy data, and then a clustering-based fault diagnosis model is developed. The least-squares support vector machine (LS-SVM) regression model is used in Han et al. (2019) to develop a chiller fault diagnosis strategy that is validated using ASHRAE data. The proposed approach is compared with two other methods using the SVM algorithm of probabilistic neural networks (PNNs). In Choi and Yoon (2021), a semi-supervised fault diagnosis approach is proposed for building automation systems using NN-based auto-encoders (AEs). An AE is a structure that transfers the input to the latent space then uses the compressed representation to produce a reconstructed version at the output. Variants of the proposed method are investigated: the residual-based approach using the error between the original and the reconstructed signal as the indication of the system status, and the latest space-based approach in which the features of the compressed representation are used for fault diagnosis.

#### Indoor environmental quality (IEQ) monitoring

IEQ monitoring continues to grow in importance, several works have demonstrated an apparent relationship between the increasing concentration of $\hbox {CO}_2$ and decreasing cognitive performance (Nejat et al. 2020; Pulimeno et al. 2020). Typically, monitoring ambient IEQ and temperature can reveal valuable information for creating a healthier, more comfortable environment for end-users. It is also a prime opportunity for energy and cost savings (Saini et al. 2020a). Thus it becomes possible, using detailed AI analytics in BAMSs, to identify various environmental problems, including air pollution, where different pollutants may affect the IEQ , such as the cleaning products, cigarettes smoke, perfumes, construction activities, water-damaged building materials, and other types of outdoor pollutants (Saini et al. 2020b). Indeed, albeit these gazes are commonly safe for end-users, their effect on human health can be dangerous if they exceed certain thresholds of exposure. To that end, an intelligent IEQ monitoring system for classifying and recognizing diverse pollutants and measuring their levels is of utmost importance (Wei et al. 2019; Muiruri et al. 2021).

Before the COVID-19 outbreak, IEQ monitoring was not a priority in public buildings, e.g. sport venues, banks, healthcare centers, academic institutions, commercial centers, restaurants, and so on. However, the fast proliferation of the corona virus and its resulting harmful effects have put IEQ in the spotlight as an important component of BAMSs. In Mumtaz et al. (2021), the authors (i) develop an IoT node including various sensors for collecting data, (ii) introduce a NN model for classifying 8 pollutants, and (iii) design an LSTM-based DL model for predicting the concentration of every pollutant and the overall IEQ. In Mad Saad et al. (2017), a pollutant recognition scheme is proposed for IEQ monitoring using different supervised ML algorithms, including MLP, KNN and linear discrimination analysis (LDA). The evaluation has been conducted in a residential building located in a rural area in China, where 5 different indoor air pollutants were considered (combustion activity, presence of chemicals, presence of food and beverages, ambient air, and presence of fragrances). While in Loy-Benitez et al. (2020b), Loy et al. introduce an ML-based scheme for detecting, diagnosing, identifying, and reconstructing abnormal observations of multivariate IEQ data in a subway station. Accordingly, a memory-gated RNN-based autoencoders (MG-RNN-AE) that can process dynamic and sequential IEQ data has been utilized.

In Cruz et al. (2020), an IEQ prediction model is developed using SVM radial basis function (SVM-RBF) and stochastic Gradient Boosting machines (SGBM). The performance of of these models has been evaluated using root mean squared error (RMSE) and $R^{2}$, and a comparison with other ML algorithms has been presented. In Taştan and Gökozan (2019), a real-time IEQ monitoring system is designed using and IoT-based e-nose and diverse ML classifiers, including SVR, generalized regression neural network (GRNN), and extreme learning machine (ELM) with Gaussian kernels. The linear correlation (LC) has been used for evaluating this framework. In Sharma et al. (2021), a cost-effective framework for IEQ prediction is introduced, where MLP and eXtream Gradient Boosting Regression (XGBR) are used for providing real-time measurements of the concentration of pollutants, i.e. $\hbox {CO}_{{2}}$ and particulate matter 2.5 ($\hbox {PM}_{2.5}$) in a set of classrooms. Moving on, an LSTM without using the forget gate (LSTM-wF) is deployed to predict the air quality at a lower complexity and increase the prediction performance.

In Alawadi et al. (2020), an indoor temperature forecasting scheme is proposed, where up to 36 ML models (pertaining to 20 different families) have been deployed. Real-world data gathered for three hours from both smart households and weather station have been used to validate this study. Similarly, in Aliberti et al. (2019), Aliberti et al. propose a smart solution for indoor air-temperature prediction, where a non-linear autoregressive neural network (NN-ARNN) has been utilized to perform short- and medium-term forecasting. This model has been then validated on both a synthetic dataset and real-world data recorded using IoT devices installed in residential buildings.

As $\hbox {CO}_{{2}}$ concentration is appropriate for measuring the IEQ quality due to its over the sensor networks, a set of frameworks have adopted it. For instance, in Taheri and Razban (2021), an ML-based IEQ monitoring approach is proposed by predicting $\hbox {CO}_{{2}}$ concentration in the academic building (campus classrooms) using demand-controlled ventilation. Various ML algorithms have been employed and compared to learn the $\hbox {CO}_{{2}}$ concentration, among them SVM, AdaBoost, RF, Gradient Boosting (GB), LR, and MLP. In a similar way, Kallio et al. (2021) propose a smart approach to forecast office indoor $\hbox {CO}_{{2}}$ concentration by adopting four ML algorithms, i.e. ridge regression (RR), DT, RF, and MLP. Moreover, a baseline to evaluate the indoor $\hbox {CO}_{{2}}$ prediction has been introduced by producing a benchmark dataset covering an entire year. Moving on, In Tagliabue et al. (2021), an ML-based IEQ monitoring approach that relies on measuring the $\hbox {CO}_{{2}}$ concentration in an academic building is proposed. Specifically, an LSTM-based RNN models and IoT sensors have been then used for monitoring the indoor conditions depending on the occupancy patterns.

Other IEQ monitoring frameworks have focused on measuring and predicting other factors, which are recommended in various countries, such as total volatile organic compounds (TVOC), formaldehyde (HCHO), and carbon monoxide (CO). For instance, in Chen et al. (2018), Chen et al. use four ML models, including SVM, Gaussian processes (GP), M5P and backpropagation neural network (BPNN) for predicting $\hbox {CO}_2$, HCHO, and TVOC in an academic building (in Singapore). In a similar way, in Lagesse et al. (2020), various ML models are utilized for predicting $\hbox {PM}_{2.5}$ in office buildings, i.e. ANN, LSTM, multiple linear regression (MLR), partial least squares regression (PLS), distributed lag model (DLM), and least absolute shrinkage selector operator (LASSO).

Other AI-big data analytics have also been used to perform additional tasks. For example, Loy et al. (2020a) introduce a variational autoencoder (VAE) coupled with convolutional layers (VAE-CNN) model to impute missing IEQ data. Accordingly, two scenarios have been adopted to evaluate the VAE-CNN algorithms: (i) a point-to-point data removal, and (ii) data intervals removing at different sampling rates. While in Kalajdjieski et al. (2020), the capability of generative adversarial networks (GANs) is exploited in combination with a data augmentation technique for overcoming the class-imbalance issue while monitoring IEQ using large-scale datasets. Table 6 outlines pertinent AI-Big data analytics frameworks introduced for monitoring IEQ and performs a comparison between them, with reference to the AI model, forecast horizon, building environment, year of appearance, method description and evaluation metrics.

**Table 6:** A summary of AI-Big data analtiycs frameworks introduced for IEQ monitoring

Ref.	AI model	Task	Building nature	Year	Description	Evaluation metrics					Others
						RMSE	MAE	MAPE	ACC	F1	
Mad Saad et al. (2017)	KNN, LDA, MLP	Pollutant ecognition	Residential	2017	Recognize 5 different pollutants using supervisedlearning				${\small \checkmark }$		
Cruz et al. (2020)	SVM-RBF,	IEQ prediction	Academic (labs)	2020	Forecast IEQ and the ideal number of occupants in a lab	${\small \checkmark }$					${\small R}^{2}$
Alawadi et al. (2020)	36 ML models	Temperature prediction	Residential	2020	Compare a set of 36 ML algorithms for temperature prediction	${\small \checkmark }$					${\small R}^{2}$
Moon et al. (2018)	SVR, GRNN, ELM	IEQ monitoring	Residential	2018	Real-Time monitoring of IEQ with an IOT-based e-nose						LC
Taheri and Razban (2021)	SVM, RF, LR, GB MLP, AdaBoost	CO$_{{\small 2}}$ concentration prediction	Academic	2021	Predict $\hbox {CO}_2$ concentration with the ability to learn nonlinearities connected with the $\hbox {CO}_2$ data	${\small \checkmark }$	${\small \checkmark }$	${\small \checkmark }$	${\small \checkmark }$		${\small R}^{2}$
Loy-Benitez et al. (2020b)	MG-RNN-AE	IEQ monitoring	Subway station	2020	Detect, diagnose, identify, and reconstruct abnormal observations of multivariate IEQ data	${\small \checkmark }$					MSE, $R^{2}$
Kallio et al. (2021)	RR, DT, RF, MLP	CO $_{{\small 2}}$ concentration prediction	Residential	2021	Forecast office indoor $\hbox {CO}_2$concentration and introduce a benchmark dataset that covers a full year.		${\small \checkmark }$		${\small \checkmark }$		
Tagliabue et al. (2021)	RNN	IEQ prediction	Academic		Monitor the ventilation rate using an IoT protocol by analyzing data with ANN communication						MSE, $R^{2}$
Aliberti et al. (2019)	ARNN	Temperature prediction	Residential	2019	Predict indoor air-temperature using synthetic data to train an ARNN						MAD, RMSD
Chen et al. (2018)	SVM, GP, M5P, BPNN	Prediction of CO$_{{\small 2}}$, TVOC and HCHO	Academic	2018	Analyze $\hbox {CO}_2$, TVOC and HCHO footprints for better predicting the IEQ			${\small \checkmark }$			$R^{2}$
Sharma et al. (2021)	MLP, XGBRLSTM-wF	IEQ monitoring	-	2021	Estimate and predict the concentration of pollutants i.e. CO$_{{\small 2}}$ and MP $_{2.5}$	${\small \checkmark }$	${\small \checkmark }$	${\small \checkmark }$			
Lagesse et al. (2020)	ANN, LSTM, MLR PLS, DLM, LASSO	Prediction of PM$_{{\small 2.5}}$	Commercial	2020	Predict PM$_{2.5}$ for IEQ monitoring in commercial office buildings	${\small \checkmark }$					
Mumtaz et al. (2021)	NN, LSTM	Classification + Prediction	Public	2021	Detect anomalies of IEQ and predict the concentration of each air pollutant	${\small \checkmark }$	${\small \checkmark }$		${\small \checkmark }$	${\small \checkmark }$	MSE

#### Security and safety

Among the major safety concerns in general and in buildings, in particular, are the fire outbreak. The prevention of and the immediate reaction to fires minimizes their consequences in terms of the people’s well-being and the financial losses. This includes the minimization of fire incidents potentials, the fast detection and extinguishment of the fire source, the effective execution of emergency evacuation, and the prompt notification of the emergency situation to the concerned authorities. Buildings are usually equipped with conventional fire alarm and extinguishment systems consisting of several sensing devices, including smoke, heat, and flame detectors, automated alarms, and water sprinklers (Zverovich et al. 2016). With the advent of big data algorithms and analytics, the fire safety in buildings can be boosted by employing buildings data to develop frameworks for fire prevention, detection, and suspension. An analysis of the advantage of utilizing ML algorithms for reliable and prompt fire detection was provided in Surya (2017) represented in their distinguished capability of black-box modeling, feature extraction, pattern recognition with high accuracy and reliability. Unlike conventional fire detection systems, ML-based models can be used to detect fire, analyze it effects, assess its risks, predict its behavior utilizing the data collected from the sensing devices.

For example, in Zhang et al. (2021), a model combining a deep belief network (DBN) and a recurrent LSTM neural network (R-LSTM-NN) was proposed for fire hazards prediction in smart cities. The proposed model was used in predicting the air quality that is then used to detect fire outbreaks based on the sensors readings of the IoT system. It shows promising potential in when data records of the IoT system are available for normal operation and scenarios of fire occurrence. In Fu (2020), a comparative study was presented using ML algorithms, namely, DT, KNN, and NNs, to develop classification models to predict failure patterns and to assess the progressive collapse potential for steel framed buildings in fire. The study aimed to develop a reliable fire assessment tool for practitioners and the developed framework demonstrated a satisfactory performance overly. In Sultan Mahmud et al. (2017), another comparative study was conducted for developing an intelligent fire detection system with early notification system in which data mining algorithms such as DTs, Bayesian networks (BayesNet), NNs, and SVM were used to develop data-driven classifiers using supervised learning. Moreover, the proposed smart fire detection system employed an edge detection model to analyze the data collected from the cameras to confirm the fire detection decision. In Huda et al. (2012), an AI-based framework was proposed to assess the thermal condition of electrical installations in buildings to prevent the potential of injuries and fire hazards using infrared images. Raw data was processed using PCA for features selection that are then used to develop an NN-based classifier to determine the condition of electrical equipment.

In Ouache et al. (2021), a fire safety assessment framework was proposed to help predict the potential fire impacts and recommending optimal fire intervention strategies in multi-unit residential buildings using NNs. Supervised learning was used to train a NN based on 5 predictors, among which are the mean of the initial fire detection (i.e., smoke detector, heat detector, visual, etc.), the action taken to fight fire (i.e., occupant response, fire department, BMS, etc.), and the performance of the BMS in fire detection and extension. The fire impact assessment covered several aspects, which are the occupant response to the incident, fire extension, fire damage, and financial losses. The proposed framework demonstrated a remarkable ability to predict fire impacts accurately, and it represented a promising solution to define and regulate fire safety strategies.

The security of the automation and management system in buildings has become more imperative due to the rapid advancement in the technologies used and the IoT systems. The industry predicts that the IoT market will grow from an installed base of 30.7 Billion devices in 2020 to 75.4 Billion in 2025 (IoT Security Foundation), which will expose them to increased risk of advanced attack vectors. According to Kaspersky Lab, nearly four in ten buildings were targeted by attacks in the first half of 2019 , and it is expected that the impact of cyberattacks on the building and construction industry will be significant in the coming years (Kaspersky). In Elnour et al. (2021), an attack detection framework for false data injection was proposed for a multi-zone HVAC system in office buildings utilizing an isolation forest (IF) algorithm. The operational data of the system’s sensor and control command signals were used to develop the detection model using semi-supervised learning. Isolation forests are characterized by the low computational requirement and capability to handle to complex and multi-variate data. They work based on pointing out anomalies using the concept of isolation, which improves the attack detection capability. Feature selection was applied to the raw data, and the study presented a comparative analysis of two models for feature reduction, which are PCA based model and a 1D-CNN-based model.

In de Assis et al. (2020), a security system for industrial IoT was proposed in which a CNN-based classifier was developed to identify distributed denial of service (DDoS) attacks in software-defined networks (SDNs). This system is based on supervised learning from the labeled network data of the IoT system. CNNs are advantageous for their high accuracy and classification performance, and powerful capability in realizing complex interdependency from multi-variate and sophisticated data. While in Aboelwafa et al. (2020), a residual-based attack detection framework was presented in which an NN-based auto-encoder was trained to profile normal system behavior. Then, non-conforming observations are identified as anomalies based on the generated residuals between the input and the output of the AE. Auto-encoders are used to learn the latent feature representation of the system using healthy operational data. They are also used for data dimensionality reduction, noise filtering, information retrieval, etc. In Yahyaoui et al. (2020), a preliminary demonstration of a ML-based intrusion detection system for data protection in healthcare centers was presented. An SVM model was developed using the labeled data of the IoT network and it demonstrated a promising performance in detecting malicious actions launched against the IoT system. The accuracy of the proposed framework was assessed based on the energy consumption in the communication network because attacks result in increased energy usage due to the increased network traffic. Table 7 highlights and compares existing AI-Big data analytics frameworks introduced to ensure security and safety in BAMSs.

**Table 7:** A summary of AI-Big data analytics frameworks proposed to ensure security and safety applications

Ref.	AI model	Task	Building nature	Year	Description	Evaluation metrics			
						ACC	MAE	MSE	Others
Zhang et al. (2021)	DBN, R-LSTM-NN	Fire hazard prediction	Smart cities	2021	Detect fire outbreak based on the sensors readings of the IoT system using supervised learning	${\small \checkmark }$			TPR, FPR Error rate
Fu (2020)	DT, kNN, NN	Fire safety assessment	Steel framed buildings	2020	Predict failure patterns and progressive collapse potential due to fire	${\small \checkmark }$			
Sultan Mahmud et al. (2017)	DT, BayesNet NNs, SVM	Fire detection	-	2017	Enhance the fire system detection capability by analyzing system data to conclude the situation	${\small \checkmark }$	${\small \checkmark }$		ACC, Precision RAE, Recall, F1
Huda et al. (2012)	PCA, NN	Inspection of electrical installations	Office	2012	Assess the thermal condition of electrical installations based on infrared images using supervised learning	${\small \checkmark }$			
Ouache et al. (2021)	NN	Fire safety assessment	Residential	2021	Investigate and assess fire protection and intervention strategies				R
Elnour et al. (2021)	IF, PCA, CNN	Attack detection	Office	2021	Detect attacks on the building management system				Precision, Recall
de Assis et al. (2020)	CNN	DDoS attack detection	Industrial	2020	Detect DDoS attacks on the SDNs of the IIoT system	${\small \checkmark }$			Precision, Recall F1 score
Aboelwafa et al. (2020)	NN	FDI attack detection	Industrial	2020	Detect FDI attacks on IIoT systems	${\small \checkmark }$		${\small \checkmark }$	FPR, TPR
Yahyaoui et al. (2020)	SVM	Intrusion detection	Hospital	2020	Detect intrusion in IoT systems				Energy

#### Occupancy detection

Occupancy data are collected by various sensors and devices in buildings to help improve the efficiency of the BAMSs in terms of energy utilization and occupants’ comfort and well-being. These include cameras, infrared sensors, and carbon dioxide detectors (Sardianos et al. 2020; Sayed et al. 2022). The data can be directly used as inputs to control and regulate some of the buildings’ equipment, such as lights, air conditioning, doors, etc. Additionally, scholars and researchers utilize big data analytics to develop approaches for analyzing and processing building occupancy data to facilitate an efficient and reliable overall building management (Sardianos et al. 2020).

In Huang and Hao (2020), a DL-based visual recognition was used to implement an occupancy detection framework utilizing CNNs. The proposed approach was used to determine the number of people present and their location to help operate demand-based HVAC systems more reliably and efficiently. It was found that the proposed approach outperforms the conventional occupancy detection systems in terms of accuracy, precision, robustness, ability to provide occupancy count, and ability of static and dynamic occupancy detection. It also requires hardware and computational considerations as CNNs have a high computational overhead. In Acquaah et al. (2020), a study was presented to estimate the occupancy count based on thermal images using CNNs for feature extraction and SVM for multi-class classification. Two well-known CNN architectures were investigated, which are the 50 layers ResNet (ResNet-50) and AlexNet using transfer learning due to their superior performance in image processing (He et al. 2016; Krizhevsky et al. 2012). In Tien et al. (2021), a computer vision-based occupancy and equipment usage detection framework was proposed to facilitate a demand driven control of the HVAC system in an office room. A multi-class region-based CNN model was developed and deployed to analyze camera images. It can predict the occupancy count, activity type (i.e. sitting, walking, etc.), and equipment usage in real-time.

A thermal-based occupancy detection approach was presented in Zhao et al. (2018) in which data-driven models were developed using SVR and RNN to predict occupancy information using the building’s properties, including indoor temperature, towards managing energy use and security monitoring in intelligent buildings. That is, the interaction of the thermal components present in the conditioned space and the necessary part of determining thermal consistency is indicated by the indoor temperature. Supervised learning was used to train the ML models using simulation data generated from Energy Plus such that the target outputs were the occupancy count. The work in Elkhoukhi et al. (2020) combined the IoT technology and Big data analytics to implement real-time occupancy detection such that data of the indoor lighting, temperature, humidity, and $\hbox {CO}_2$ levels were used to predict the status of the building occupancy. Two models were developed and tested, one using LDA and the other using vertical hoeffding tree (VHT) for offline and online occupancy detection, respectively. Additionally, in Fatema and Malik (2021), feature extraction and correlation analysis were performed on indoor sensors data (i.e., temperature, $\hbox {CO}_2$ level, light intensity, humidity) and then particle swarm optimization was used to train an NN-based occupancy detection model. It demonstrated improved classification performance compared to conventional NNs optimized using the back-propagation algorithm.

In Wu and Wang, a ML-based model was proposed to improve the operation of the BAMS due to the shortcomings of infrared sensors for stationary occupancy. The model predicted the occupancy status based on multiple statistical features of the signals acquired by the infrared sensors. ML algorithms were investigated, among which SVM demonstrated the best performance due to its ability to capture complex and nonlinear functions, and its efficacy in handling high dimensional data. In Huchuk et al. (2019), a comparative analysis was presented for occupancy forecasting using ML algorithms based on thermostat data. The prediction model was intended to optimize the operation of the air conditioning system, such that both the present and the future occupancy information is taken into consideration. It was found that RF algorithm outperformed the LR, the Markov model, the hidden Markov model (HMM) and the RNN, which is based on the bagging technique in which multiple models on different subsets of the training dataset are developed, then their predictions are combined to conclude the final output of the RF model. However, occupancy forecasting is only dependable when the building does not exhibit rapid and random fluctuations in the user profile, which is generally the case for residential buildings.

In Razavi et al. (2019), a comparative study was presented for the utilization of supervised ML algorithms such as SVM, RF, KNN, and NNs to estimate and predict occupancy information in residential buildings based on power meters data. It was found that the reliability of occupancy prediction is lower for the larger forecast horizons. In Feng et al. (2020), a DL-based approach is proposed combining a CNN and a bidirectional LSTM network for occupancy detection in houses based on electrical data of advanced metering infrastructures (AMIs). The data essentially contain readings of electric current, voltage, and power that are processed by the CNN for spatial feature extraction. Using supervised learning, the extracted features are then fed to the BiLSTM network to solve a binary classification problem to identify the occupancy condition in real-time. The proposed framework demonstrated improved performance when compared to other ML and DL based models due to its ability to interpret the spatial and contextual features of the data. However, since detailed occupancy information are mostly not recorded and hence not available, supervised learning-based approaches that are based on such a detailing in the data (i.e., people count) can be impractical and difficult to implement using actual building data. While in Pešić et al. (2019), a LSTM network-based framework was proposed to perform occupancy detection and forecasting as well as data analytics based on Bluetooth positioning and WiFi utilization data of the IoT infrastructure in a multi-story residential building. The network data were pre-processed to extract the information of the occupancy of the apartments, then used to develop the LSTM network to predict and forecast occupancy condition and patterns in the different spaces of the building. The proposed work demonstrated the effective fusion of Bluetooth and WiFi data as well as the successful deployment of NN-based data analytics using wireless networks data for occupancy detection application. Table 8 summarizes the relevant AI-Big data analytics frameworks developed to detect occupancy profiles.

**Table 8:** A summary of AI-Big data analytics models used for occupancy detection

Ref.	AI model	Detection basis	Building nature	Year	Description	Evaluation metrics			
						ACC	MAE	MSE	Others
Huang and Hao (2020)	CNN	Surveillance cameras	Office	2020	Detect the number and location of occupants	${\small \checkmark }$			Relative error
Acquaah et al. (2020)	CNN, SVM	Thermal cameras	–	2020	Estimate the number of people present based on thermal images	${\small \checkmark }$			
Tien et al. (2021)	CNN	Vision cameras	Office	2021	Predict equipment use and occupancy count & activity in real time	${\small \checkmark }$			Precision, Recall, F1
Zhao et al. (2018)	SVR, RNN	Temperature sensor	Office	2018	Detect the number of occupants based on indoor thermal properties				Error rate
Elkhoukhi et al. (2020)	LDA, VHT	Indoor sensors	Office	2020	Predict the status of occupants’ presence	${\small \checkmark }$			
Fatema and Malik (2021)	NN	Indoor sensors	Office	2021	Predict occupancy condition in an office room	${\small \checkmark }$			TPR, FPR, Precision, Recall, F1, MCC
Wu and Wang	SVM, kNN, DT RF, NN	Infrared sensor	–	2021	Provide accurate predictions of the occupancy status based on motion detectors	${\small \checkmark }$			
Huchuk et al. (2019)	LR, HMM, MM, RF, RNN	Thermostat data	Residential	2019	Forecast the occupancy information	${\small \checkmark }$			
Razavi et al. (2019)	kNN, SVM, NN, RF	Energy meter	Residential	2019	Estimate and predict occupancy information	${\small \checkmark }$			Precision, AUROC
Feng et al. (2020)	CNN, BiLSTM	Smart meters	Residential	2020	Predict real-time occupancy status based on data of electrical signals	${\small \checkmark }$			Precision, Recall, F1, TNR F1, Training time
Pešić et al. (2019)	LSTM	Bluetooth and WiFi devices	Residential	2019	Predict, forecast, and analyze occupancy information using wireless networks data	${\small \checkmark }$			RMSE and Edit Distance on Real Signals

#### Water usage management

Almost all kinds of buildings are users of water, although the cost of water and sewer services varies from area to area and can become a significant expense. Worse, in areas where there is a shortage of water, it is not only a big expense, but an imperative to conserve. Therefore, it becomes of significant importance to bring the monitoring of water levels and switching points of all wet applications in buildings to the BAMS. Furthermore, water monitoring systems can benefit from the advancement of AI and ML technologies for improving their performance (Sun and Scanlon 2019). Typically, by harnessing the power of AI-big data analytics, it is possible to maximize information and data available and hence make better decisions while enhancing service delivery and reducing costs (Rahim et al. 2020).

**Fig. 5:**
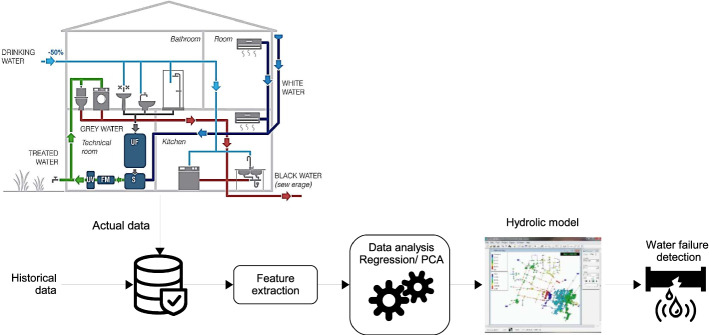
Flowchart of water usage monitoring system used to detect water leaks and optimize water consumption (Jenny et al. 2020)

In this context, using IoT water meters with wireless connectivity (Bluetooth, LoRaWAN, etc), it becomes relatively easy to install water meters within the building. These can be as simple as pulse style meters that can easily be integrated into a BAMS. Moving on, adopting AI-big data analytics to analyze data from water meters has become crucial for optimizing the management of water resources and sustaining growth and development. Accordingly, various AI-big data analytics frameworks have recently been proposed with the aim of (i) processing complex nonlinear water data, (ii) forecasting water demand, (iii) predicting water meter failures, or (iv) monitoring the quality and temperature of the water. Fig. 5 illustrates the flowchart of a water usage monitoring system used to detect water leaks and optimize water consumption (Jenny et al. 2020).

In Altunkaynak and Nigussie (2017), water demand prediction is conducted by first using multiplicative season algorithm (MSA) to extract pertinent information from water meter records and also capturing periodicity and converting nonstationary signals into stationary signals. Following, their output is fed into an MLP for accurately predicting water demand. The RMSE and Nash-Sutcliffe coefficient of efficiency have been adopted to evaluate the prediction performance of the learning model and its ability to extend prediction lead time. Shine et al. (2018), diverse ML models are used for predicting water consumption in an agricultural building based on analyzing data collected from a remote monitoring system. Thus, RF, ANN, SVM, and CART decision tree (CDT) algorithms were trained to predict water consumption, where a backward sequential variable selection was adopted for excluding variables adding low predictive power along with a hyper-parameter tuning with nested cross-validation for calculating the prediction accuracy for each model. In a similar way, in Smolak et al. (2020), three ML algorithms are implemented and compared for predicting water usage, i.e. RF, SVM, ARIMA. The water consumption data augmented with end-users occupancy patterns were used to improve the prediction accuracy. A novel approach to process and correlate between occupancy and water usage time-series was introduced. This framework was validated on 51 days of water consumption readings and over 7 million occupancy patterns from urban areas.

On the other hand, by using AI-big data analytics, it is also possible to monitor water quality and hence improve water resources management plans. In Chen et al. (2020), 10 ML models are deployed to water quality prediction (WQP). Specifically, DT, NB, LR, LDA, completely-random tree (CRT), KNN, SVM, RF, and deep cascade forest (DCRF) have been trained using water data from a hydro-electric power (HEP) plant, including pH, DO, CODMn, and NH3-N to forecast water quality. The precision, recall, F1 score, and weighed F1-score (wF1) have been selected to evaluate the prediction performance of the ML algorithms. In Roccetti et al. (2019), Roccetti et al. develop an ML-based classifier, which is personalized for predicting the failure of a water meter. Typically, an RNN model is deployed for (i) processing 15 million of readings collected from 1 million of mechanical water meters, and (ii) extracting relevant patterns representing the complex phenomenon of defective water meters. This has helped in achieving more than 80% accuracy in detecting failures.

In Wang et al. (2018), water demand of urban areas is predicted using gravitational search algorithm (GSA) and backtracking search algorithm (BSA) with ANN with regard to various weather parameters. While in Antunes et al. (2018), four ML models are selected to predict water demand, including ANN, RF, SVM and KNN, through the analysis of real-world data from two Portuguese water utilities. Moving forward, a weighted parallel strategy for combining multiple ML algorithms is introduced to improve the prediction performance. Moreover, additional data related to weather, seasonality, and feature extraction (forecast window of time-series data) are also analyzed. In Nasser et al. (2020), the water demand prediction is performed using an LSTM model based on analyzing data gathered from intelligent IoT water meters. A cloud platform has been used to store water consumption records, enabling near real-time data streaming and storing. The performance of LSTM has been then compared to those of SVR and RF. Similarly, in Du et al. (2021), Du et al. propose an LSTM model that combines discrete wavelet transform (DWT) and PCA to forecast daily urban water demand. Therefore, after smoothing the outliers of water demand time-series, noise components are removed using DWT and pCA. Following, the LSTM network is deployed to predict urban water demand using the outputs of DWT and PCA. Table 9 presents the main AI-Big data analytics frameworks proposed for water management in buildings.

**Table 9:** A summary of AI-Big data analtiycs models for water monitoring

Ref.	AI model	Task	Building nature	Year	Description	Evaluation metrics					Others
						RMSE	MAE	MAPE	ACC	F1	
Altunkaynak and Nigussie (2017)	MLP	Water demand prediction			Predict water demand using MSA-MLP and compare its DWT-MLP	${\small \checkmark }$					CE
Zubaidi et al. (2018)	GSA-ANN, BSA-ANN	Water demand prediction	Residential	2018	Predict water demand using heuristis algorithms, ANN and weather variables	${\small \checkmark }$					
Chen et al. (2020)	DT, NB, LR, LDA,CRT, KNN, SVM, RF, CRF	WQP	HEP plant	2020	Predict water quality using different water paranmetersi.e. pH, DO, CODMn, and NH3–N					${\small \checkmark }$	wF1
Shine et al. (2018)	RF, NNN, SVMCDT	Water consumption prediction	Agricultural	2018	Predict water consumption using a backward sequential variable selection and parameter tuning			${\small \checkmark }$	${\small \checkmark }$		
Smolak et al. (2020)	RF, SVM, ARIMA	Water consumption prediction	Residential	2020	Predict water consumption using consumption records and occupancy patterns	${\small \checkmark }$	${\small \checkmark }$	${\small \checkmark }$	${\small \checkmark }$		
Antunes et al. (2018)	ANN, RF, SVM KNN	Water demand prediction	Public	2018	Reliable prediction while no significant anomalies of the data used during training are reported	${\small \checkmark }$		${\small \checkmark }$			$R^{2}$
Roccetti et al. (2019)	RNN	Predicting water meter failures	-	2019	Predict water meter failures using 15 million of readings				${\small \checkmark }$	${\small \checkmark }$	AUC, CM
Nasser et al. (2020)	LSTM	Water demand prediction	Public and residential	2020	Predict energy demand by analyzing data gathered from smart IoT water meters and stored in the cloud	${\small \checkmark }$	${\small \checkmark }$	${\small \checkmark }$			
Du et al. (2021)	LSTM-DWT-PCA	Water demand prediction	Public and residential		The outputs of DWT and PCA are fed into an LSTM network to predict water demand	${\small \checkmark }$		${\small \checkmark }$			EVS, $R^{2}$

### Evaluation metrics

Evaluation metrics are used to measure the performance of the model in terms of the quality of its output as per what is expected. For AI applications, there are various types of metrics that can be used based on the subject matter. That is, the outputs of an AI model can take two forms, which are categorical variables (Cvars) and quantitative variables (Qvars). For instance, the outputs of classification models represent categorical variables in which the input data are classified into different groups or classes which are characterized by a unique label or value such as detection problems, recommender systems, etc. Each observation can be placed in a single category, and the categories are mutually exclusive. Hence, the performance of the model depends on its ability to correctly classify the observations to their respective categories/groups.

On the other hand, quantitative variables represent numerical values that exhibit quantitative characteristics. Regression models have quantitative variables as outputs such as forecasting and estimation models in which the AI model is used to represent the mapping between the independent variable(s)—i.e., the input(s)—and the dependent variable(s)—i.e., the output(s)—. In this case, the quality of the model is measured by the closeness of the model’s outputs to the ideal expected values. Table 10 presents a summary of the common metrics used to evaluate AI models.

**Table 10:** A summary of the common evaluation metrics of AI models

Metric	Description	Values type	Application	Formula
Relative error (RE)	The absolute error between the actual and estimate values of a variable to its estimate value	Qvars	Regression	$\left| \left( y - {\hat{y}}\right) /{\hat{y}}\right|$
Relative change	The amount of the absolute difference as a fraction of the variable’s reference value	Qvars	Regression	$\left( \left| y - y_\mathrm{ref}\right| /y_{\mathrm{ref}} \right) \times 100\%$
Mean absolute error/difference (MAE or MAD)	Measures the absolute difference between predicted and actual variables describing the same phenomenon.	Qvars	Regression	$\frac{1}{m} \sum _{i=1}^m \left| {\hat{y}}_{i}-y_{i}\right|$
Mean absolute percentage error (MAPE)	the MAE expressed in percentage.	Qvars	Regression	$\text {MAE} \times 100\%$
Median Absolute Error (MedAE)	Measures the median absolute error between predicted and actual values.	Qvars	Regression	$\text {Median}\left( \left| {\hat{y}}_{i}-y_{i}\right| \right)$
Mean squared error/difference (MSE or MSD)	Measures the average difference between the predicted and actual values.	Qvars	Regression	$\frac{1}{m} \sum _{i=1}^m ({\hat{y}}_{i}-y_{i})^2$
Root mean squared error/difference (RMSE or RMSD)	The square root of the MSE to interpret the error in the same unit of the variable.	Qvars	Regression	$\sqrt{\frac{1}{m} \sum _{i=1}^m ({\hat{y}}_{i}-y_{i})^2}$
Root mean square percentage error (RMSPE)	Represents the RMSE expressed in percentage	Qvars	Regression	$\text {RMSE} \times 100\%$
Normalized root mean squared error (NRMSE)	Refers to the normalized RMSD to facilitate the comparison between variables with different scales.	Qvars	Regression	$\text {RMSE}/(y_{max} - y_{min})$
R-squared ($\hbox {R}^2$)	Measures the fit quality of a regression model/function by representing the proportion of the variance for a dependent variable in terms of independent variable(s).	Qvars	Regression	$1 - \left( \sum _{i=1}^m ({\hat{y}}_{i}-y_{i})^2/\sum _{i=1}^m ({\bar{y}}_{i}-y_{i})^2\right)$
Theil U1 index	Measures the relative accuracy between the actual and predicted results.	Qvars	Regression	$\sqrt{\sum _{i=1}^m\left( {\hat{y}}_{i}-y_{i}\right) ^2/\sum _{i=1}^m y_{i}^2}$
Theil U2 index	Measures the quality of the predicted results.	Qvars	Regression	$\sqrt{\frac{1}{m}\sum _{i=1}^m\left( {\hat{y}}_{i}-y_{i}\right) ^2}/\left( \sqrt{\frac{1}{m}\sum _{i=1}^m y_{i}^2} + \sqrt{\frac{1}{m}\sum _{i=1}^m {\hat{y}}_{i}^2}\right)$
Accuracy (ACC)	Measures the closeness between the predicted values and the targets.	Cvars	Classification	$\mathrm ( {TP} + {TN} ) / ({TP} + {TN} + {FP} + {FN})$
Error rate (ERR)	Measures the proportion of the false predictions in total predictions.	Cvars	Classification	$\mathrm ({{{FP}} + {{FN}}})/({{{TP}} + {{TN}} + {FP} + {FN}})$
Precision (PPV)	Measures the closeness the set of predicted result	Cvars	Classification	$\mathrm {{TP}}/({{TP} + {FP}})$
Recall or True positive rate (TPR)	Measures the proportion of correct positive (TP) predictions in the true positive class.	Cvars	Classification	$\mathrm {{TP}}/({{TP} + {FN}})$
False-positive rate (FPR)	Measures the proportion offalse positive (FP) predictions in the true negative class.	Cvars	Classification	$\mathrm {{FP}}/({{FP} + {TN}})$
True-negative rate (TNR)	Measures the proportion of correct negative (TN) predictions in the true negative class.	Cvars	Classification	$\mathrm {{TN}}/({{TN} + {FP}})$
False-negative rate (FNR)	Measures the proportion of false negative (FN) predictions in the true positive class.	Cvars	Classification	$\mathrm {{FN}}/({{TP} + {FN}})$
F1-score	Measures the harmonic average of the precision and recall	Cvars	Classification	$\mathrm 2({{PPV}\times {TPR}})/({{PPV}+{TPR}})$
Matthews correlation coefficient (MCC)	Measures the quality of binary classifications.	Cvars	Classification	$\frac{{\mathrm{TP}}\times {\mathrm{TN}} - {\mathrm{FP}}\times {\mathrm{FN}}}{\sqrt{({\mathrm{TP}}+ {\mathrm{FP}})({\mathrm{TP}} + {\mathrm{FN}})({\mathrm{TN}}+{\mathrm{FP}})({\mathrm{TN}} + {\mathrm{FN}})}}$
Prediction interval coverage probability (PICP)	Evaluates whether the actual value $\alpha$ is within the prediction interval limits,$\alpha _i = 1$ if it lies within the prediction interval, $\alpha _i = 0$ otherwise	Qvars	Regression	$\frac{1}{N}\sum ^{N}_{i=1}$,
Prediction interval normalized average width (PINAW)	Measures the width of the prediction interval with $L_i$ and $U_i$ being the lower and the upper boundaries, respectively, and *E* is the difference between the maximum and the minimum actual values.	Qvars	Regression	$\frac{1}{NE}\sum ^{N}_{i=1} \left( U_i -L_i\right)$
Precision recall curve (PRC)	Used to present the trade-off between precision and recall using different thresholds.	Cvars	Classification	–
Receiver operating characteristic curve (ROC)	Used to present the trade-off between the false positive rate and true positive rate using different thresholds.	Cvars	Classification	–
Area under the ROC (AUROC)	Represents the area under the ROC curve and the greater the value, the better the classification performance	Cvars	Classification	–
Cross validation (CV)	A resampling procedure to evaluate the generalization ability of an AI model	Cvars and Qvars	Classification and Regression	–
Confusion matrix	A tabulated representation of classification of results of the algorithm under study.	Cvars	Classification	–

## Critical discussion and current challenges

A truly smart building combines a BAMS with intelligent data analytics software that offers helpful insights for maintenance, service, and efficiency opportunities. Typically, these tools together offer benefits for building owners, such as: (i) providing a high-level, system-wide big data capture of the entire operations, (ii) ensuring air quality control and a healthier building environment, (iii) saving energy and energy consumption during off-peak or low occupancy periods, (iv) eliminating waste from everyday system usage through intelligent sensor data, (v) offering guidance for performance improvements for individual assets, (vi) addressing equipment that really needs repair and not just those on a fixed schedule that don’t need to be serviced, and (vii) offering advanced automation capabilities and actionable results. In addition to these benefits, the cost savings related to the use of smart data analytics can be significant.

However, various gaps specific to each application field of AI-big data analytics are identified. Among them, more effort should be put to efficiently carrying out text analytics on operators’ work-order logs; and identifying (i) the information to derive, (ii) the text-mining methods to adopt, and (iii) the efficient approaches to convey the information to the operator and visualize it. Moreover, another challenging issue concerns the use of virtual metering, where a limited number of works were dedicated to virtual meter development (Kim et al. 2021; Wilcox 2020) despite its significance in helping operators for understanding plant-to-zone water and energy flows and ranking their operational decisions, such as identifying and evaluating faults.

The HVAC prognostic and failure prediction is another application that is still very challenging, where limited research activity was conducted to target this challenge. In fact, developing prognostics models is valuable for (i) predicting the time-to-failure, (ii) avoiding global failures in key BAMS components (e.g. boilers, chillers, pumps, fans, etc.), and preventing disruptions in building services. In addition, there are new challenges from emerging BAMSs that need to be addressed, e.g. data benchmarking, big data security and privacy, scalability and interoperability, real-time big data intelligence and knowledge transfer.

### Data quality issues

Usually, raw data gathered from BAMSs can have some data quality problems, including (i) outliers, (ii) noise, (iii) inconsistent data, (iv) duplicate data, and (v) missing values. Data pre-processing techniques are deployed to overcome these issues, such as formatting, cleaning, and resampling. Formatting aims at converting the raw data into appropriate formats to ease the application of ML algorithms, while cleaning refers to removing or replacing missing samples (Zhang et al. 2021). Lastly, resampling can be applied based on the requirements of ML algorithms. Typically, it can be (i) a down-sampling to reduce data redundancy, foster the processing and improve the accuracy; or (ii) an up-sampling that helps increase the amounts of data to train data-hungry ML models, especially DL algorithms (Elnour et al. 2022). To that end, because of the high requirements for data quality set by ML models, developing novel strategies to improve the quality of BAMS recorded data by creating additional data with enhanced quality or augmenting existing datasets is a crucial challenge.

### Data scarcity and data benchmarking

The different applications of BAMSs necessitate extensive historical data to train the AI-big data analytics, especially those based on DL algorithms before they can be used reliably. However, large-scale data might not be available for some reason or can not be recorded representatively and sufficiently in a short time when we study newly-built environments. Fortunately, the problems addressed within each specific AI-big data analytics task in BAMSs illustrate some similarities. This could be justified by the fact that the different application tasks, despite studying distinct problems, use the same data-driven algorithms which are validated on slightly similar datasets collected from different kinds of buildings and devices. This has opened opportunities for using knowledge transfer and transfer learning to overcome the lack of datasets in some situations, e.g. sports facilities.

On the other hand, collecting and benchmarking data represents the most significant challenge so far when applying AI-big data analytics in BAMSs, especially for the case of large buildings, i.e. sports facilities, commercial centers, industrial buildings. Many tasks require annotated datasets to train AI models and validate them. Indeed, developing and validating new data-driven algorithms require recording and annotating large-scale datasets, especially when using DL algorithms that are notoriously data-hungry (Kučera and Pitner 2018). Improving the performance of BAMSs does not rely only on the selection of AI algorithms but also on the quality and parameters of datasets used to train them.

For instance, labeled and accurate anomaly detection datasets are needed for developing new automatic anomaly detection solutions. Similarly, development and validation of occupancy detection algorithms require repositories of building occupancy profiles with concurrent ground-truth people counts. In this context, to further improve the performance of BAMSs, public and open-access datasets are needed for different application fields (e.g., load forecasting, anomaly and fault detection, demand response, occupant-centric controls, IEQ monitoring, water monitoring, etc.) for assessing the AI-big data analytics algorithms developed by the AI Community (Park et al. 2019; Francisco et al. 2020).

From another hand, successful sustainability strategies to overcome this issue could be via (i) incentivizing buildings/facilities managers for participating in benchmarking campaigns and surveys organized for different application fields, and (ii) encouraging the AI community in organizing data benchmarking competitions and challenges.

### Security and privacy preservation

The nature of data collected in BAMSs introduces new challenges in data analytics, i.e. security and privacy preservation, in which traditional technologies can not deal with. Using encapsulated protocols and IP-based communication, BAMSs are more and more connected to corporate networks and also remotely accessed for management reasons, both for emergency and convenience purposes. However, security and privacy preservation have not been set as a primary concern when designing these protocols. Therefore, most of the BAMSs are being operated with sub-standard or non-existent security implementations, and mainly rely on ensuring security by obscurity. In this line, there has been recently a move to address the shortfalls of security and privacy preserving implementations in BAMSs (Stamatescu et al. 2020; Ashaj and Erçelebi 2020). However, the definition of the new threats against BAMSs, and identification of these threats is still a field that is exceptionally lacking.

Moreover, another critical concern about security in BAMSs is related to the fact that buildings’ data is valuable not only for managers and other BAMS competitors, as it is attached to the control of buildings’ equipment. Typically, it could be significantly critical to end-users if manipulated. To that end, sharing data in most BAMSs has limited the buildings’ intranet. Also, any attempts to extract this data to cloud data centers can result in severe security risks, considerably higher costs for the appropriate security systems or both (Lv et al. 2021; Himeur et al. 2022).

### Scalability and interoperability

An important issue of BAMSs is the inherent lack of scalability and interoperability. This is because each BAMS manufacturer has its own proprietary data protocol that requires the development and maintenance of various processes and integrations. Moreover, BAMS vendors usually have competing products and thus are incentivized to make their data inaccessible to third parties (Png et al. 2019; Tang et al. 2020). Therefore, the interoperability is a legitimate concern for making efficient smart buildings as it refers to the ability of all the systems inside a building to communicate with one another. Specifically, with the actual proliferation of intelligent building technologies designed and manufactured by a plethora of companies, there is a need to make them communicate universally for promoting their deployment inside residential, commercial, industrial, and office buildings (Ozturk 2020; Miori et al. 2019). Put differently, as smart buildings need to meet energy and water efficiency, adequate indoor environmental conditions, high comfort levels, and economic goals set by building managers/users, they require the use of a highly-connected building automation system in which different parts can efficiently communicate with one another and adjust to changes in the environment. However, while numerous buildings are equipped with excellent systems, they often lack a combined monitoring system for lighting, climate control, water monitoring and blinds that could facilitate efficiency measures (Schachinger et al. 2017).

The BAMS community makes great efforts to develop and deploy communication protocols that ensure interoperability, such as Modbus, KNX, LonWorks, and Protocol 3964R (Merz et al. 2018). For instance, LonWorks and KNX are interoperable open standards as they can be used together. However, integration concerns may arise (Tang et al. 2020). The technologies’ popularity can hinder interoperability potential in BAMSs, as the example with LonWorks, the market leader in the United States. In contrast, KNX—widely used in Europe—has yet to impact (Merz et al. 2018). Additionally, the installation and operation costs can pose severe integration limitations. Indeed, many contractors or system integrators that provide off-the-shelf solutions are less concerned about whether the integration is successful.

### Real-time big data intelligence

Collecting and analyzing data in real-time are of significant importance while designing powerful and efficient BAMSs. The first step towards this is by adopting a real-time sub-metering, which helps in tracking track utility costs by building region (floor, room, etc), by the tenants, by individual facility equipment (e.g., HVAC, lighting), etc. Therefore, granular utility sub-metering data provides the essential tools for monitoring energy costs/performance, water consumption/waste. This will result in accurately identifying usage anomalies, enabling data-driven portfolio analysis, etc. Although the value of real-time sub-metering is unquestioned, most of the BAMSs are still unable to provide the real-time data monitoring, which delays decision-making measures and hence reduces then the quality of efficiency and optimization operations.

## Case studies

This literature review established several applications of AI big data analytics for buildings in terms of energy management, load forecasting, water management, FDAD, or IEQ monitoring. In this section, we present their deployment for energy-related applications given the continuous rise in energy consumption worldwide of the buildings sector under the global energy dilemma and the energy optimization potential of BAMSs, given the increasing concerns about energy efficiency in buildings. More specifically, the case studies handle two of the lead causes of energy waste in buildings, which are (i) system faults and equipment malfunctioning, and (ii) poor management and regulation of the buildings’ systems (Alsalemi et al. 2022; Elnour et al. 2022, 2020). The first two case studies present strategies for energy anomaly detection that can be due to both or either of the former causes. They demonstrate the deployment of two different methods: unsupervised and supervised learning. The last case study presents the use of AI data analytics to establish reliable and efficient regulation of HVAC systems, given that those are considered major energy consumers in buildings (energy.gov, Elnour et al. 2022; Fadli et al. 2021).

### Unsupervised AI-based energy anomaly detection

**Fig. 6:**
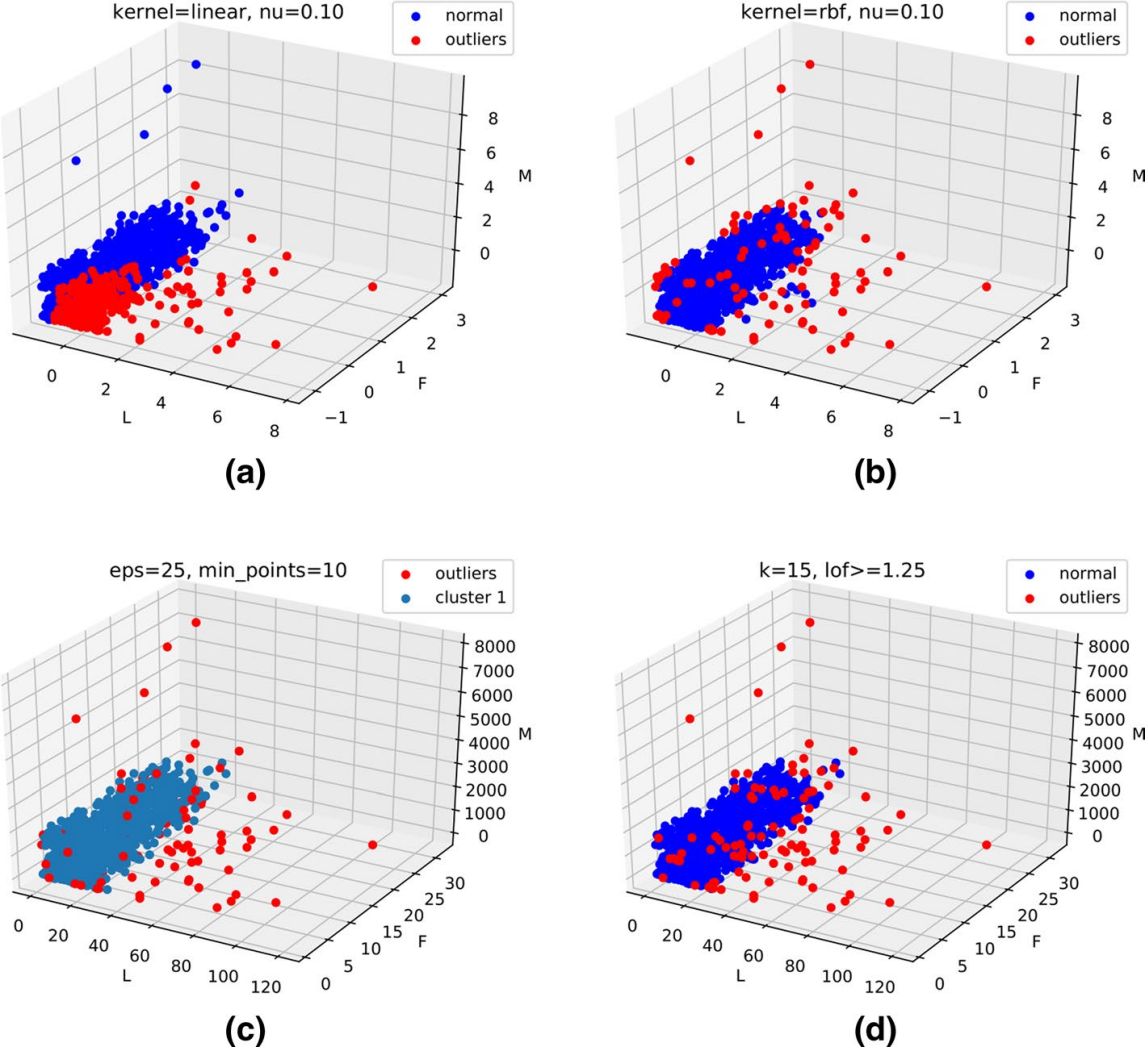
Energy consumption anomaly detection in residential buildings using a) OCSVM with linear kernel, b) OCSVM with Gaussian kernel, c) DBSCAN and d) LOF

This section presents an example of using unsupervised ML algorithms for detecting abnormal energy consumption (Himeur et al. 2022). Therefore, four algorithms are considered, namely (i) OCSVM with linear kernel, (ii) OCSVM with Gaussian kernel, (iii) DBSCAN, and (iv) LOF. They have been applied on the Dutch residential energy dataset (DRED), which incorporates electricity consumption, occupancy patterns and ambient conditions of a typical household (in the Netherlands). Figure 6 portrays the scatter plot of energy footprints in which normal and abnormal patterns are identified using the aforementioned approaches. It has been clearly seen OCSVM (with linear kernel) detects more energy samples that fall outside the inlier region, which refer to consumption anomalies. While by using OCSVM (with a Gaussian kernel), the number of samples that fall inside the inlier region has been reduced because of its separation capability introduced by the hyperplane generated using the Gaussian kernel. From another side, LOF and DBSCAN help detect abnormal patterns with almost the same efficiency as OCSMV (with the Gaussian kernel), and only a slight difference has been registered in classifying a few numbers of samples.

### Supervised AI-based energy anomaly detection

**Fig. 7:**
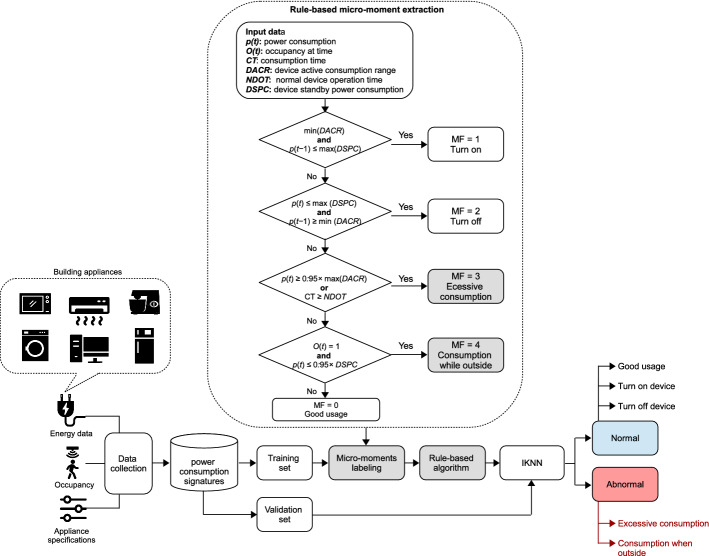
Block diagram of the supervised ML solution used to detect abnormal energy consumption in office buildings

Supervised ML algorithms excelled in detecting abnormal energy usage, although they require labeled energy data. To that end, in Himeur et al. (2021), a micro-moment-based approach is introduced to cluster energy footprints of an office building (at Qatar University) into five classes with reference to the energy consumption, occupancy patterns and appliance operation specifications. These classes are named ”class 0: good usage”, ”class 1: turn on appliance”, ”class 2: turn off appliance”, ”class 3: excessive consumption” and ”class 4: consumption while outside”. Following, an improved KNN model is developed and used to learn abnormal energy usage using this annotated data. Fig. 7 illustrates the flowchart of the micro-moment based scheme used to extract and learn intent-driven moments of energy consumption. Typically, energy micro-moment features *MF* are extracted based on analyzing occupancy profiles (*O*) and power consumption (*p*) of each device in reference to device active consumption range (*DACR*), device operation time (*DOT*) and device standby power consumption (*DSPC*). Then, the appliance operation parameters are called, including *DACR*, *DOT* and *DSPC*. Table 11 presents an example of different appliance parameter specifications that are used in the rule-based algorithm to extract power consumption micro-moments (Himeur et al. 2022).

**Table 11:** Power consumption specifications for different home appliances

Appliance	DOT	DACR (watts)	DSPC (watts)	Appliance	DOT	DACR (watts)	DSPC (watts)
Air conditionner	15 h 30 min	1000	4	Washing machine	1 h	500	6
Microwave	1h	1200	7	Light	8 h	60	0
Oven	3h	2400	6	Television	12 h 42 min	65	6
Dishwasher	1h 45 min	1800	3	Refrigerator	17 h 30 min	180	0
Laptop	12 h 42 min	100	20	Desktop	12 h 42 min	250	12

**Fig. 8:**
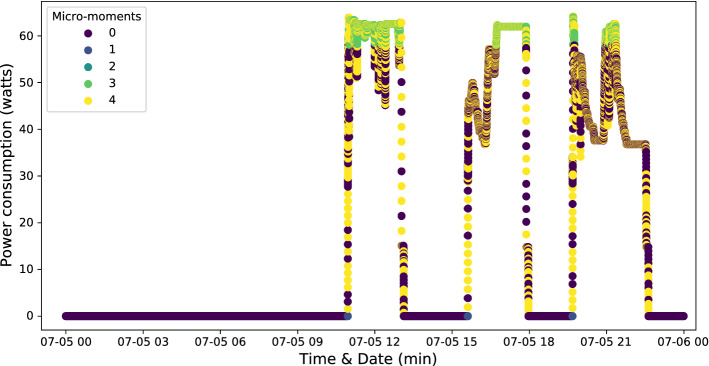
Scatter plot of energy micro-moments identified using IKNN (Himeur et al. 2021)

To have a clear view of how abnormal energy consumption is distributed over the time, the scatter plot of energy consumption profiles of a television is illustrated in Fig. 8. Accordingly, the corresponding normal and abnormal energy patterns are detected using an IKNN model and micro-moment analysis. Because this approach uses a supervised learning with regard to occupancy data, it has the capability of identifying new consumption anomalies that correspond to the absence of the end-users when the television is on (this abnormality can be extended to other devices that require the presence of the user during their operation, e.g. the air conditioner, heater, fan, etc.). Detecting such abnormalities was not doable if an unsupervised ML model was deployed, in which only energy patterns were analyzed.

### Energy and performance optimization for sports facilities

**Fig. 9:**
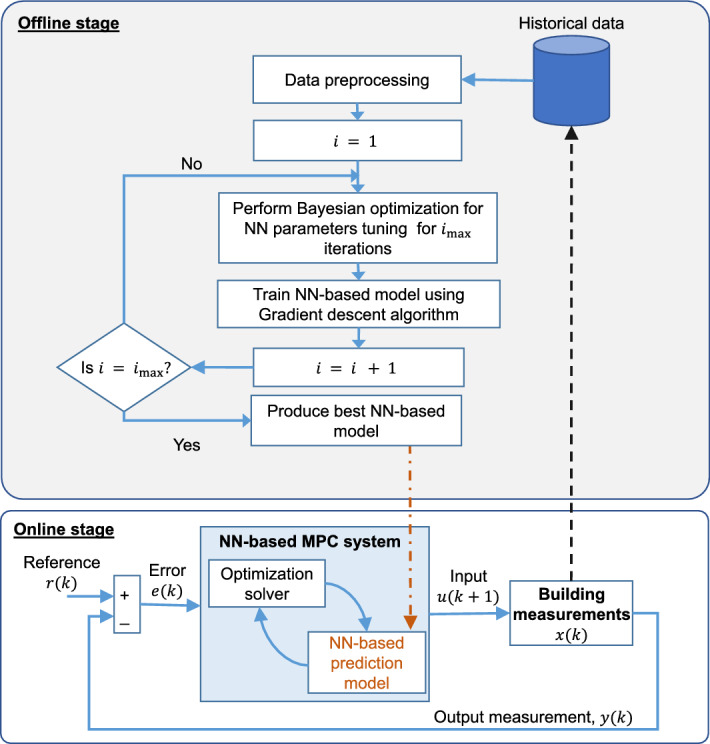
Block diagram of the NN-based MPC framework for sports facilities energy and performance optimization

In light of the increased global energy demand and its associated environmental impacts, the management and optimization of sports facilities are becoming imperative as they are characterized by high energy demand and occupancy profiles. This case study demonstrates the application of the model predictive control (MPC) theory and NNs for energy and performance management of sports facilities. Figure 9 presents the proposed NN-based MPC framework. The work is carried out using the building information model of a sports hall in the sports complex of Qatar University using EnergyPlus and practical data for model calibration. MPC systems are robust as they allow integrated dynamic optimization that accounts for the future system behavior in the decision-making process. NNs are advantageous for their ability to represent complex functions with high accuracy.

The NN-based dynamic prediction model aims to express and capture the behavior of the building operation over time given its states *x*(*k*) (i.e., power usage, thermal comfort, indoor and outdoor air properties, etc.) and its inputs (i.e., HVAC system settings). The NN-based prediction model is:

1 $$\begin{aligned} F = \text {Train\_NN}\left( {x}(k+1),[x(k), u(k+1)]\right) , \end{aligned}$$

and the prediction is computed by:

2 $$\begin{aligned} {\hat{x}}(k+1) = F\left( x(k), u(k+1)\right) . \end{aligned}$$

The optimization of the NN’s hyper-parameters was performed using the Bayesian optimization algorithm which keeps track of past iterations to find better choices for the next set of hyper-parameters to evaluate (Andonie 2019).

**Fig. 10:**
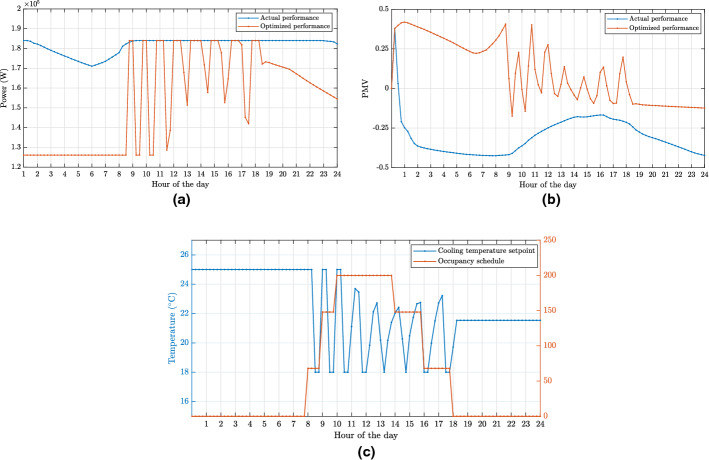
The performance of the NN-based MPC system for energy and performance optimization in the sports hall in Qatar University sports complex

The MPC system consists of an optimizer and an NN-based prediction model of the building operation, and based on the system output $y(k) \subset x(k)$ and its reference value *r*(*k*) (i.e., power usage and thermal comfort level), the HVAC system settings for temperature setpoints and dampers positions are determined (i.e., *u*(*k*)) using numerical optimization to achieve tracking. When compared to routine performance, the proposed approach was able to achieve significant energy reduction and adequate thermal comfort levels as demonstrated in Fig. 10. Energy savings of around 15% was observed, which was approximated by evaluating the relative change in the total energy consumption in the two settings for the scenario under study, that is, the relative difference between the areas under the two power curves in Fig. 10a. Considerations about the NN model performance, tuning of the MPC settings, and optimization sub-optimality or failure are essential during the design and implementation phases of the proposed framework.

#### Improved computational sustainability model for sports facility management

**Fig. 11:**
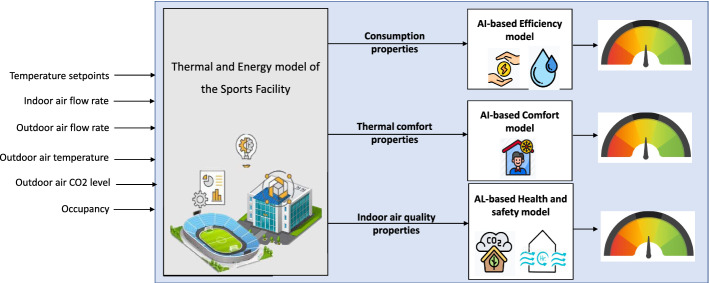
Block diagram of the improved CUSP model with efficiency-comfort-health model

The computational urban sustainability platform (CUSP), developed at Cardiff University, is an immersive decision support tool built to deliver a powerful urban analytics and enable interactive monitoring and inform decision making through a web interface. It can be used to promote co-simulation across disciplines, and predict future scenarios towards a sustainable future operation and urban Intelligence [Computational Urban Sustainability Platform (CUSP)]. The CUSP model can be improved to include three integrated models, which are (1) energy-water efficiency, (2) health, safety, and wellbeing, and (3) comfort as demonstrated in Fig. 11.

**Fig. 12:**
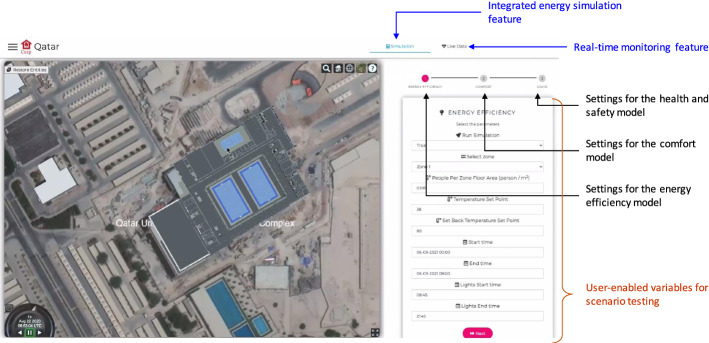
The web interface of the expanded and improved CUSP platform with the three integrated models for efficiency, comfort, and health & safety

The improved CUSP model integrates an energy simulation tool that is used to generate data of the particular scenario under consideration for data analytics for quality monitoring and planning purposes. It contains three AI-based models developed to assess each of the three aspects of the facility operation, which are efficiency and sustainability, health and safety, and users’ thermal satisfaction. Through the web interface shown in Fig. 12, the integrated simulation tools will enable facility managers to evaluate the possible scenario in terms of the HVAC system settings, and occupancy and operation schedules towards achieving a reasonable trade-off between those three aspects prior to applying them in the facility.

## Future directions

### Multimodal data analysis

Due to the advancement of today’s sensing and mobile technologies, various modalities of data can be easily and effectively gathered using different and advanced means. Thus, it is now possible to record and process big data about environmental satisfaction levels of buildings’ occupants in real-time and non-invasive manners (Plageras et al. 2018). Buildings’ end-users naturally react to ambient environmental conditions for minimizing any environmental stress, increasing their comfort based on their autonomic nervous systems and expressed by different poses, which can effectively influence different building operation parameters (Amato et al. 2018). Therefore, it becomes of utmost importance to develop tools for enhancing the interdisciplinary knowledge (i.e. AI, IoT, big data, DL, computer vision) when managing building operations. This helps significantly advance building indoor environmental control and sensing technologies as a function of human bio-signals (i.e. physiological signals) and poses.

Analyzing multi-modal data helps BAMSs in boosting workplace productivity and optimising office spaces, which in turn cutting costs and increasing revenues for companies. Moreover, data generated from these systems could be used to reduce the spread of viruses and other diseases inside buildings, increasingly important since the outbreak of Covid-19 (Sun and Zhai 2020). For instance, in Ding et al. (2020), investigate the collective contagion of the COVID-19 virus inside indoor environments (i.e. healthcare facilities and public vehicles) along with the engineering control against virus spread with ventilation systems.

### In-situ sensor calibration in BAMSs

Sensors are key players in helping BAMSs to attain expected efficiency and automation. However, they are affected by continuous failures and degradation over time. To that end, in-situ sensor calibration plays a crucial role in calibrating different BAMS working sensors (i.e., physical sensors) and avoiding significant errors for reliable results when it is deployed to large-scale building sensor networks (Yu and Li 2015). Most of the studies opted for the conventional periodical calibration as a solution to overcome sensor degradation and failure; however, this is impractical and difficult for various sensors. By contrast, virtual in-situ calibration (VIC) can be a good alternative since it relies on mathematically extracting the characteristics of essential aspects involved in a calibration, such as the uncertainty quantification, benchmark establishment, and environment assessment (Yoon and Yu 2018). Moreover, because BAMSs need digitally enhanced data-rich environments, virtual sensors offer reliable and informative sensing contexts for operational datasets in BAMSs. More specifically, in-situ virtual sensors help develop the counterparts of target physical sensors in the field. Therefore, they can provide extra data related to residuals between physical and virtual sensors for for deployment in data-driven modeling, diagnostics and analytics (Koo et al. 2022).

### Smart building digital twins

The increasing amounts of data generated by BMAMs, and the need for new methods to leverage it, have motivated scientists to investigate new strategies. One promising solution is using the digital twins (DT) paradigm, which assumes a complete cohesion and integration between the visual and physical worlds. Typically, DT can deliver considerable benefits to the BAMSs and the built environment in general by helping bring together static and dynamic data from various sources (in 2D/3D models) and assisting in making effective and informed decisions. Moreover, it combines the knowledge from the physical and digital worlds by collecting real-time data from the physical environments and provides a real-time understanding of buildings’ performance (Delgado and Oyedele 2021). Besides, despite the gradual exploration of digital twinning within the fields of building information modeling (BIM) and cyber-physical systems (CPS), available tools and techniques need to be considered in the next level of integration (technologies and procedures). This is to (i) provide DTs with more adaptability and more cohesion over the managed information and (ii) extract more value from our virtual models (Shahzad et al. 2022).

### Transfer learning

Specifically, transfer learning has recently been proposed as solution that can be investigated for the case of buildings with poor information data (Himeur et al. 2022). Put simply, data and knowledge of already existing buildings (or old buildings) with rich energy usage records, water management data, occupancy patterns, IEQ monitoring footprints and and ambient environmental conditions can be used. Therefore, various frameworks have been introduced for target energy forecasting (Gao et al. 2020; Li et al. 2021), anomaly detection of energy consumption (Liang et al. 2018; Xu et al. 2021), fault diagnosis of energy systems (Liu et al. 2021; Zhu et al. 2021), HVAC fault detection (Dowling et al. 2020), IEQ monitoring (Tariq et al. 2021), indoor occupancy detection (Khalil et al. 2021), etc.

### Blockchain

Due to the security and privacy issues that are still open in BAMSs, blockchain is considered as a promising solution that provides the digital trust. It can function as a permanent, cloud-based and digital ledger of activities between different users and partners (Nawari and Ravindran 2019; Liu et al. 2021). Also, blockchain can operate as a distributed, single source of shared truth and has the possibility of becoming the top-system for recording all transactions. Therefore, its deployment in BAMSs aims at (i) tracking and validating changes (e.g. security and surveillance, access control, etc.), (ii) monitoring HVAC activities, (iii) recording property transfers, and (iv) detecting occupancy patterns (Siountri et al. 2020). Additionally, it can help manage intelligent buildings and IoT devices with renewable energy, e.g. wind and solar. For example, suppose a facility is in a two-way energy communication with the grid. In that case, blockchain can make it more secure and easier to develop a digital record of energy-in and energy-out transactions (Tiwari and Batra). On another side, as the global market of building automation exceeds $120 billion, smart contracts can be utilized for automating warranties and providing refunds when IoT-connected devices or equipment do not perform as expected (Himeur et al. 2022).

Overall, there are numerous potential applications of blockchain in BAMSs, although the principal advantages are data is easy to access, is secure, and can not be corrupted. Specifically, data stored in the blockchain database can be easily and quickly reviewed, even though it is managed by distinct entities, which results in an accurate and fast data analysis (Nawari and Ravindran 2019). Moving forward, blockchain helps in streamlining processes and lowering costs through reducing and/or eliminating those dreaded manual operations, especially in public buildings, sports facilities, and commercial centers. This could be adapted to almost any process, including preventive maintenance, work orders, environmental health, and safety planning, and space management (Nawari and Ravindran 2019).

Only for energy management in smart buildings, blockchain has found diverse applications. For instance, in Van Cutsem et al. (2020), use a blockchain-based approach to cooperate energy management of multiple end-users in smart-buildings, where smart-contracts have been utilized to allow decentralizing community energy management. In Mukherjee et al. (2021), a smart energy management solution is safeguarded with blockchain and hence ensures judicious generation, uniform distribution and shielded monitoring along with guaranteed security and privacy of the havoc data. In Tiwari and Batra, blockchain is introduced for enabling the reparation of smart buildings-cyber physical systems. Moving on, decentralized and flexible access control using smart contracts is developed for smart and large commercial buildings in Bindra et al. (2021). This solution has been proposed as an alternative to inefficient, unsystematic, and human-intensive access control schemes usually used in these buildings. While the widespread implementation of blockchain is still a long way off, it is also challenging to deploy this technology reliably and widely in BAMSs. This research area needs to be further investigated in the near future. This promising new technology could benefit the other tasks of smart buildings, i.e. water management, IEQ monitoring, occupancy detection, etc.

### Cyber-security standards for BAMSs

While using AI in BAMSs represents a powerful asset, it also presents some data security and privacy concerns and problems with the regulations. Typically, AI-driven BAMSs involve the deployment of lower-cost sensors (both wired and wireless) and the adoption of cloud, fog, edge, and/or hybrid computing architectures, increasing cyber risks. To that end, the need for a sound cybersecurity strategy has become crucial for promoting secure remote BAMSs. Data flows must be planned and monitored, possibly making it necessary to use one-way data diodes. On the other hand, BAMSs integrate heterogeneous sensing, computation, and control capabilities. They combine cyberspace with the physical world to develop cyber-physical systems. However, the security of BAMSs is significantly threatened by software/hardware failures and/or cyber/physical attacks. For example, sensor failures can engender false detection of abnormal energy/water consumption behaviors and result in actuator misbehavior.

To handle the above issues, privacy and security protection mechanisms should be enforced. This is possible by providing recommendations to the building automation community, e.g., the data protection directive 95/46/EC (Tokarski) suggests recommendations for supporting the security of the implementation of smart metering and smart using data controllers. Addressing these recommendations can enable moving to fully harmonized data protection environments and improving security measures in BAMSs. Moreover, different cyber security standards can be used to secure BAMSs by addressing the cybersecurity for operational technology in automation and control systems, such as ISA/IEC 62443 series (Bicaku et al.) and ASHRAE 135 series (BACnet) (Tang et al. 2020).

### Self-learning for long-term building operation

Self-learning ML models are key to realizing the BAMS in the long-term building operation. The systems built upon these models have recently gained industry recognition and market share as they are based on using a ”user-friendly” technology (Cortiços 2019). Typically, self-learning, also called self-supervision, is an emerging technology that helps develop computationally efficient, low-cost, autonomous, and self-supervised ML algorithms (Kaklauskas et al. 2019). For example, for energy management, a self-learning control scheme assists in assessing the energy flexibility of buildings, in addition to guaranteeing robustness, scalability, and adaptability. Moreover, automated self-learning systems have promising perspectives when they are to integrate demand-response strategies for effective home-energy management systems (Bampoulas et al. 2019).

### Edge analytics for BAMSs

With the advancement of BAMSs and the latest generation of IoT devices, data acquisition from multiple types of equipment has become much easier in today’s buildings. Real-time access to this data helps in better managing facility operations, sustaining efficiency, and lowering costs. However, as most BAMSs are only implemented using cloud computing, real-time data analysis may not be guaranteed. To overcome this issue, open software platforms hosted on edge nodes in close proximity to the building’s IoT devices can enable access to the building data and advanced analytics deployed on these platforms in real-time. In this context, edge computing employs the processing power of IoT devices for filtering, pre-processing, aggregating and storing recorded data, and actions can then be performed in real-time using adequate analytical algorithms. This is because edge computing enables resolving bandwidth and latency problems and reducing response time. Following, the filtered data could be transmitted to the cloudlet platforms for aggregation and enrichment, and running of complex analytics (Sharma et al. 2018).

Thus, various use cases where edge computing and the IoT can efficiently be utilized in BAMSs are emerging, among them fault diagnosis, which helps to (i) find patterns in sensor data representing equipment failures, anomalies, or degraded performance; (ii) detect abnormal energy consumption, e.g. if the lighting or HVAC systems are activated too early or operate too late with regard to the actual occupancy schedules; and (iii) identify correlations across different types of data, which are essential to infer the factors impacting energy consumption (e.g. the patterns related to weather, age of facilities, etc). Overall, open edge software platforms combine multi-protocol connectivity and the ability to aggregate data from multiple sources and facilitate the task of advanced analytics in turning this data into actionable information that can be used to improve the overall operational efficiency buildings (Petri et al.).

## Conclusion

This paper carried out a comprehensive overview of the application of AI-big data analytics in BAMSs to conduct different tasks, including energy forecasting, fault and anomaly detection, water monitoring, and IEQ monitoring. The pros and cons of AI models within the unsupervised, supervised, semi-supervised, and reinforcement learning categories have been identified. Moreover, it concluded that supervised learning algorithms excelled well in performing the diver BAMS tasks, but their performance always relies on the availability of annotated data and its accuracy. Unsupervised learning models with no prior knowledge can address this issue with less efficiency.

It was demonstrated in this framework that technologies of ML, IoT, and new connectivity capabilities have a critical role in shaping the future of BAMSs. With building owners and facility managers focusing heavily on improving energy efficiency and increasing cost savings, features like advanced fault detection and diagnostics, energy analytics, IEQ monitoring, and water management are becoming critical. The growing interest devoted to developing intelligent analytics in BAMSs has been highlighted by the increasing number of works and studies proposed in the literature to address several challenges. In the coming years, data analytics is expected to expand the capabilities of intelligent building technologies, spurring further advancements in building automation systems and equipment standards while minimizing the environmental impact of commercial buildings.

The AI-big data analytics technology is up-and-coming to BAMSs. However, it faces various challenges for achieving market penetration, including legal, regulatory, security and privacy preservation, interoperability and scalability, and competition barriers. Additional research initiatives, investigations, projects, and collaborations should be considered a primary requirement for showing if the technology can reach its absolute power, prove its commercial viability, and lastly, be adopted in the mainstream.

## Abbreviations

A3C: Asynchronous advantage actor-critic
AC: Actor-critic
AE: Auto-encoder
AHU: Air handling unit
AMI: Advanced metering infrastructures
ANN: Artificial neural network
AR: Auto-regressive
Adaboost: Adaptive Boosting
BAAKR: Bagged auto-associative kernel regression
BAMS: Building automation and management system
BBN: Bayesian belief networks
BBRT: Bootstrap bagging of regression trees
BDT: Binary decision tree
BESN: Bagged echo state network
BMCDT: Binary multiclass-classification decision tree
BN: Bayesian networks
BNN: Bagging neural network
BPNN: Back propagation neural network
BRT: Bagged regression tree
BSA: Backtracking search algorithm
BT: Bagged tree
BiLSTM: Bidirectional long short-termmemory
CAGR: Compound annual growth rate
CART: Classification and regression tree
CCTV: Closed-circuit televi-sion
CDT: CART decision tree
CIFG: Coupled input and forget gate
CNN: Convolutional neural network
CPS: Cyber-physical system
CRBM: Conditional restricted Boltzmann machines
CRF: Conditional random fields
CRGP: Compact regression Gaussian process
CRT: Completely-random tree
CUSP: Computational urban sustainability platform
ConvLSTM: Convolutional LSTM
DBN: Deep belief network
DBSCAN: Density-based spatial clustering of applications with noise
DCRF: Deep cascade forest
DDPG: Deep deterministic policy gradient
DDoS: Distributed denial of service
DFNN: Deep feed forward neural networks
DL: Deep learning
DNN: Deep neural networks
DQL: Deep Q-learning
DRED: Dutch resi-dential energy dataset
DRL: Deep reinforcement learning
DT: Decision tree
DWT: Discrete wavelet transform
EBT: Ensemble bagging tree
ELM: Extreme learning machine
FCM: Fuzzy C-means
FCRBM: Factored conditional restricted Boltzmann machines
FFNN: Feed-forward neural network
GAM: Generalized additive models
GBM: Gradient boosting machine
GBRT: Gradient boosting regression tree
GLRM: Generalized linear regression model
GPRM: Gaussian process regression model
GRU: Gated recurrent units
GSA: Gravitational search algorithm
GSD: Generated sampled data
HCA: Hierarchical cluster analysis
HEP: Hydro-electric power
HMM: Hidden Markov model
HVAC: Heating ventilation and air conditioning system
ICT: Information and communication technology
IEQ: Indoor environmental quality
Isomap: Isometric feature mapping
KNN: K-nearest neighbors
KPCA: Kernel principal component analysis
LDA: Linear discriminant analysis
LOF: Local outlier factor
LR: Linear regression
LR: Logistic regre-ssion
LSSVR: Least squares support vector regression
LSTM: Long short-term memory
MC: Monte Carlo
MCSVM: Multi-class support vector machines
MDA: Multiple discriminant analysis
MDS: Multidimensional scaling
MLP: Multi-layer perceptron
MLR: Multiple linear regression
MPC: Model predictive control
MSA: Multiplicative season algorithm
MetaFA: Metaheuristic firefly algorithm
NB: Naive Bayes
NILM: Non-intrusive load monitoring
NN-SAE: Neural network-based-supervised auto-encoder
NN: Neural network
OCSVM: One-class support vector machine
PCA: Principal component analysis
PG: Policy gradient
PLS: Partial least square
PPO: Proximal policy optimization
QDA: Quadratic discriminant analysis
QL: Q-leaning
QR: Quantile regression
RBDT: Regression binary decision tree
RBFNN: Radial basis function neural network
RBM: Restricted Boltzmann machines
RF: Random forest
RFT: Regression fitting
RL: Reinforcement learning
RNN: Recurrent neural networks
RT: Regression tree
ResNet: Deep residual network
SAE: Memory-gated RNN-based autoencoders
SAE: Sparse autoencoders
SARIMA: Seasonal autoregressive integrated moving average
SARSA: State-action-reward-state-action
SDN: Software-defined networks
SGPR: Stepwise Gaussian processes regression
SSL: Semi-supervised learning
SSNN: Semi-supervised neural network
SVM: Support vector machine
SVR: Support vector regression
TRL: Tradi-tional reinforcement learning
TSVD: Truncated singular value decomposition
TTS: Text-to-speech
VAE: Variational autoencoders
VHT: Vertical hoeffding tree
WQP: Water quality prediction
XGBM: Extreme gradient boosting machine
XGBoost: EXtreme gradient boosting
mLSTM: Multip-licative LSTM
t-SNE: t-Distributed stochastic neighbor embedding

## Footnotes

1 https://www.researchandmarkets.com/reports/4774956/big-data-analytics-market-in-the-energy-sector.

2 https://www.energy.gov/sites/prod/files/2017/03/f34/qtr-2015-chapter5.pdf.

3 https://www.iotechsys.com/markets/industries/building-automation/.

4 https://www.dialog-semiconductor.com/products/industrial-edge-computing/smartserver-iot-edge-server.

5 https://www.lynxspring.com/technology.
